# A role for BDNF- and NMDAR-induced lysosomal recruitment of mTORC1 in the regulation of neuronal mTORC1 activity

**DOI:** 10.1186/s13041-021-00820-8

**Published:** 2021-07-12

**Authors:** Dany Khamsing, Solène Lebrun, Isabelle Fanget, Nathanaël Larochette, Christophe Tourain, Vincent de Sars, Maia Brunstein, Martin Oheim, Damien Carrel, François Darchen, Claire Desnos

**Affiliations:** 1 Saints-Pères Paris Institute for the Neurosciences, Université de Paris, Centre National de la Recherche Scientifique UMR 8003, 45 rue des Saints Pères, 75006 Paris, France; 2 Université de Paris, Centre National de la Recherche Scientifique, INSERM, B3OA, Paris, France, Ecole Nationale Vétérinaire d’Alfort, B3OA, Maisons-Alfort, France; 3 Wavefront-Engineering Microscopy Group, Sorbonne Université, INSERM S968, CNRS UMR7210, Institut de la Vision, Paris, France; 4 Service de Psychiatrie Infanto-Juvénile, Centre Hospitalier de Gonesse, 2 Boulevard du 19 mars 1962, 95500 Gonesse, France

**Keywords:** mTOR, Endo-lysosomes, NMDA receptors, BDNF, Synaptic plasticity, Optogenetics

## Abstract

Memory and long term potentiation require de novo protein synthesis. A key regulator of this process is mTORC1, a complex comprising the mTOR kinase. Growth factors activate mTORC1 via a pathway involving PI3-kinase, Akt, the TSC complex and the GTPase Rheb. In non-neuronal cells, translocation of mTORC1 to late endocytic compartments (LEs), where Rheb is enriched, is triggered by amino acids. However, the regulation of mTORC1 in neurons remains unclear. In mouse hippocampal neurons, we observed that BDNF and treatments activating NMDA receptors trigger a robust increase in mTORC1 activity. NMDA receptors activation induced a significant recruitment of mTOR onto lysosomes even in the absence of external amino acids, whereas mTORC1 was evenly distributed in neurons under resting conditions. NMDA receptor-induced mTOR translocation to LEs was partly dependent on the BDNF receptor TrkB, suggesting that BDNF contributes to the effect of NMDA receptors on mTORC1 translocation. In addition, the combination of Rheb overexpression and artificial mTORC1 targeting to LEs by means of a modified component of mTORC1 fused with a LE-targeting motif strongly activated mTOR. To gain spatial and temporal control over mTOR localization, we designed an optogenetic module based on light-sensitive dimerizers able to recruit mTOR on LEs. In cells expressing this optogenetic tool, mTOR was translocated to LEs upon photoactivation. In the absence of growth factor, this was not sufficient to activate mTORC1. In contrast, mTORC1 was potently activated by a combination of BDNF and photoactivation. The data demonstrate that two important triggers of synaptic plasticity, BDNF and NMDA receptors, synergistically power the two arms of the mTORC1 activation mechanism, i.e., mTORC1 translocation to LEs and Rheb activation. Moreover, they unmask a functional link between NMDA receptors and mTORC1 that could underlie the changes in the synaptic proteome associated with long-lasting changes in synaptic strength.

**Supplementary Information:**

The online version contains supplementary material available at 10.1186/s13041-021-00820-8.

## Introduction

Long-lasting changes in synaptic strength, in particular those associated with the late phase of long term potentiation (L-LTP), are thought to represent the cellular substrate of memory encoding [1, 2]. Many studies have shown that de novo protein synthesis is essential to the formation of different form of neuronal plasticity including LTP [2–5]. Some mRNA translation has been demonstrated to occur in neuronal sub-compartments remote from the cell body such as dendrites and dendritic spines [3, 6–10]. The whole machinery needed for mRNA translation is present in these compartments and local protein synthesis may be elicited by synaptic inputs [5, 11]. The maintenance of protein homeostasis related to neuronal plasticity also requires proteasomal and autophagy-lysosomal degradation [12, 13]. A major pathway that controls this balance is the mechanistic target of rapamycin complex 1 (mTORC1) [14]. This ubiquitous complex, possessing a serine/threonine kinase activity, is a master controller of several anabolic and catabolic processes, that integrates many environmental and intracellular inputs such as nutrients, energy levels and growth factors. Several forms of synaptic plasticity including LTP depend on mTORC1 [5, 15, 16]. LTP induction triggers phosphorylation of mTORC1 substrates which is accompanied by synthesis of some synaptic proteins [8–10, 17]. On the other hand, rapamycin, a well-characterized allosteric inhibitor of mTOR kinase, blocks LTP [8, 16, 18]. Furthermore, genetic inactivation of several regulators or effectors of mTORC1 such as TSC1/2, p70S6K1/2, FKBP12, and 4EBP2 interferes with synaptic plasticity or long-term memory encoding [19–22]. However, despite the remarkable progress made recently on understanding mTOR signaling in health and diseases in specific tissues, little is known about the regulation of mTORC1 signaling in brain [14, 23].

mTORC1 is a dimeric complex consisting of the protein kinase mTOR, the subunits Raptor, mLST8, DEPTOR and PRAS40 [5, 14, 24]. In different cell types, mTORC1 activation leads to an increase in protein synthesis and cell growth and to a decrease in catabolic processes. On the contrary, upon starvation, mTORC1 and anabolic processes are silenced while macroautophagy and lysosome biogenesis are activated to restore amino acid levels [14]. mTORC1 controls mRNA translation via at least two well-established downstream targets: p70S6 kinase (p70S6K), which phosphorylates several translation factors including the ribosomal protein S6 (pS6), and repressor proteins of eukaryotic initiation factor 4E (eIF4E) termed 4E-BPs [5]. By doing so, mTORC1 promotes the assembly of the eIF4F initiation complex and regulates the translation of a set of 5’-capped mRNAs. Active mTORC1 suppresses autophagy by phosphorylation-dependent Atg13–ULK1 inhibition and impairs the nuclear translocation of TFEB, thus inhibiting the transcription of lysosomal and autophagy related genes [14, 25].

The main upstream pathways that regulate mTORC1 activity involve phosphatidylinositol-3-kinase (PI3K), Akt, and AMPK, responding to signals such as growth factors, energy, oxygen levels that converge on the tuberous sclerosis complex (TSC), which acts as a GTPase Activating Protein (GAP) for the small GTPase Rheb (Ras homolog enriched in brain). Inactivation of TSC1/2 results in increased levels of Rheb-GTP that directly binds to mTOR to power its activity [26, 27]. mTORC1 activation also requires amino acids, cholesterol [28] and glucose [29] that trigger mTORC1 recruitment to lysosomal structures through Raptor, where the complex can interact with its activator Rheb. Thus, activating Rheb and recruiting mTORC1 to cell compartments where Rheb is concentrated represent two arms of mTORC1 activating mechanism. How mTORC1 is translocated to late endocytic compartments (LEs) has been the focus of recent studies that revealed the role of multimeric complexes [14]. One is a heterodimer made of Rag-GTPases that can interact with Raptor [30–32]. The guanine nucleotide status of Rag GTPases is regulated by certain amino acids and the LE v-ATPase and involves different lysosomal scaffolds, such as Ragulator [33–35], GATOR1 [36, 37], Sestrin2 [38], Folliculin (FLCN/FNIP2) [39–41], and the lysosomal transporter SLC38A9 [28, 42, 43]. A Rag-independent pathway, sensitive to glutamine and asparagine, mediated by Arf1, has also been described [44, 45].

As mentioned before, mTORC1 plays an important role in brain and especially in hippocampus where it controls postnatal synaptic plasticity via regulation of synaptic protein translation or degradation in an activity dependent manner [15, 46–49]. This localized rapamycin-sensitive translation is crucial for the remodeling of dendritic spines that accompanies LTP [4, 50, 51]. Furthermore, dysregulation of the mTORC1 pathway is associated with synaptic defects involved in a set of characteristic neuronal diseases [14, 23, 52] such as Alzheimer’s disease, autism spectrum disorder, mood disorders or familial epilepsies [53].

In neurons, mTORC1 signaling is activated not just by nutrients and insulin but also by specific inputs, including neurotransmitters and their membrane receptors (e.g., glutamate and acetylcholine) and brain-derived neurotrophic factor (BDNF) acting on TrkB receptor [15, 17, 54], a major activator of the PI3K/Akt/TSC/Rheb pathway. Activity-dependent release of BDNF [55, 56] is important for LTP especially in hippocampus [57, 58]. NMDA receptors (NMDAR) activation affects neuronal activity and plasticity through different pathways, one of which involves mTORC1 [8, 15, 46] and activated NMDARs can trigger BDNF secretion [55, 56].

However, how NMDARs activate mTORC1 remains obscure. The molecular mechanism of neuronal activation of mTORC1 and especially the role of LEs in this mechanism, has not been clarified yet [23, 53]. In this study, we addressed the localization of mTORC1 in hippocampal neurons under non-stimulated conditions or upon induction of LTP, and developed pharmacological and optogenetic strategies to promote or restrict the translocation of mTORC1 to LEs in order to probe the contribution of this translocation on mTORC1 activity.

## Materials and methods

### Antibodies

For western blotting, the following antibodies were used: rabbit anti-Phospho-S6-ribosomal protein (Ser240/244, Cell Signaling 1:1000), rabbit anti-Phospho p70S6K (Thr 389, Cell Signaling 1:1000), mouse anti-actin (Sigma 1:1000), goat anti-rabbit or mouse peroxidase conjugated secondary-antibodies (Sigma, 1:10,000). For immunofluorescence, were used: mouse anti-Map2B (Millipore 1:1000), chicken-anti-Map2B (PhosphoSolutions, 1:500), rabbit anti-Phospho-S6 ribosomal protein (Ser240/244, Cell Signalling, 1:800), rabbit anti-mTOR (Cell Signaling, 1:300), rat anti-LAMP1 (1D4B; Developmental Studies Hybridoma Bank, University of Iowa, USA, 1:1500), mouse anti-FLAG (Sigma,1:1000) and mouse anti-myc 9B11 (Cell Signalling, 1:1500), appropriate fluorescent Alexa-conjugated secondary antibodies (Invitrogen, Carlsbad, CA, 1:2500).

### DNA constructs

pmCherry-C1 was provided by Clontech. pmCherry-Rheb plasmid was obtained as follows: the coding sequence of Rheb was amplified by PCR from pMXs-EGFP-Rheb-IP plasmid (a gift from Shinya Yamanaka; Addgene plasmid # 13831) to add XhoI and BamHI restrictions sites and was then subcloned into pmCherry-C1 plasmid using these sites. pmCherry-RhebQ64L mutant plasmid was done using Q5 site-directed mutagenesis (New England Biolabs) with the following primers: F 5’-TCAGATCTCGAGGGCCTC-3’ and R 5’-GTCCGGACTTGTACAGCTC-3’. pmyc-Raptor and pLJM1-Flag-Raptor-Rheb15 (Flag-Raptor-Rheb-TS) were a gift from David Sabatini (Addgene plasmids # 1859 and # 26634, respectively). pCRY2-mCherry-Raptor was obtained by amplifying the coding sequence of Raptor by PCR from myc-Raptor to add BsrGI and XbaI restriction sites and by subcloning the PCR product into pCRY2PHR-mCherry-N1 (a gift from Chandra Tucker, Addgene plasmid # 26866) using these sites. pCIB1-myc-Rab7 was obtained as follows: first, CIB1 coding sequence was PCR amplified from pCIBN(deltaNLS)-pmGFP (Addgene plasmid # 26867) and subcloned into peGFP-Rab7 plasmid (a gift from Dr Suzanne Pfeffer, Stanford University) using NheI and AgeI restriction sites. Then, GFP sequence was replaced with myc sequence using Q5 site-directed mutagenesis with the following primers: 5’-TCGGAGGAGGACCTACGAGCCTCTAGGAAGAAAG-3’ and 5’-GATTAGCTTCTGCTCGACCGGTACATGAATATAATC-3’. All constructs were verified by sequencing.

### Animals

Mice were treated in accordance with guidelines for the care and use of laboratory animals (US National Institutes of Health) and the European Directive number 86/609 (EEC Council for Animal protection in Experimental Research and Other Scientific Utilization). The experiments were approved by Paris Descartes University ethics committee (Permit Number: CEEA34.FD.047.11.). Mice were euthanized by cervical dislocation and embryos by decapitation. Adult rats were euthanized by CO_2_ inhalation and neonates by decapitation.

### Cultured cells

HeLa cells were seeded in 12 well plates at a density of 35,000 cells per cm^2^ and incubated at 37 °C, with 5% CO2 in Dulbecco's Modified Eagle Medium supplemented with 4.5 g/L glucose, glutamine (DMEM High glucose GlutaMAX™-I, Gibco) 10% fetal calf serum (FCS, Gibco) and 0.1% penicillin/streptomycin (Gibco).

Primary hippocampal cell cultures were prepared from E16 embryonic Swiss mice (Janvier Labs), as previously described [59]. Briefly, hippocampi were dissected out in pre-chilled (4 °C) Neurobasal medium (Gibco) using a stereomicroscope, and the meninges carefully removed. Hippocampi from 10–12 embryos were collected, washed twice with pre-chilled HBSS (Gibco), and incubated for 10–12 min in 250 µg/ml trypsin (Sigma) with 250 µg/ml DNAse (Roche) and 50 mM Hepes–NaOH pH 7.2 diluted in HBSS in a 37 °C water-bath. Cells were then dissociated by trituration and plated at a density of 25,000/cm^2^ on poly-D-Lysine-coated Petri dishes (100 µg/ml) (Falcon, Thermo Fischer Scientific) or poly-d-Lysine-coated glass coverslips when used for immunofluorescence. Plating culture medium consisted of Neurobasal medium supplemented with 2% B27 (Life Technologies, Gaithersburg, MD), 2 mM GlutaMAX™-I (Invitrogen), penicillin 5 U/mL and streptomycin 2.5 µg/mL. Cells were maintained at 37 °C in 5% CO_2_. After 6 to 24 h, plating medium was replaced by glial cell-conditioned medium (CM). Neurons were maintained for 15–21 days in vitro (DIV). Synaptic morphology was verified as previously described [60].

Primary cultures of glial cells were prepared from PDN4 Sprague–Dawley rats (Janvier Labs). Cortices were dissected out and processed as previously described [59]. Cells were plated in 75 cm^2^ flasks in DMEM High glucose GlutaMAX™-I supplemented with 10% FCS and 0.1% penicillin–streptomycin. Cells were let to grow at 37 °C in 5% CO_2_ until they were sub-confluent (80%) and passaged to 1:2 in the same medium. Passages #1 and #2 were used to prepare conditioned medium (CM) as followed: DMEM medium was replaced by pre-warmed Neurobasal medium supplemented with 2% B27, GlutaMAX™-I and 0.1% penicillin–streptomycin for 24 h to 48 h. The conditioned medium was collected, filtered and stored at − 20 °C and used once to feed primary hippocampal neurons.

### Transfections

Hippocampal neuron cultures were transfected with endofectin® (Tebu-Bio) between 11 and 15 DIV. For one coverslip (2 cm^2^ well), 1 µL of endofectin diluted in 50 µL Neurobasal medium containing 0.4 µg of plasmid DNA (adjusted to 400 ng with pcDNA3 if needed) was added to 300 µL of neuronal growth medium. The rest of growth medium was stored. Cultures were incubated in transfection mix for 3 h at 37 °C, 5% CO_2_; transfection medium was then replaced with the stored neuron growth medium. HeLa cells were transfected using lipofectamine 2000 (Invitrogen) 24 h after seeding, with 0.4 µg of DNA plasmid (100 ng of CIB1-myc-Rab7; 100 ng of CRYII-mCherry-Raptor and adjusted to 400 ng with pcDNA3) for 3 h and the transfection medium was then replaced by culture medium.

### Drug treatments, neuronal stimulation and viability test

Neuronal stimulation was performed in ACSF buffer (ACSF w/o Mg^2+^): 125 mM NaCl, 2 mM CaCl_2_, 2,5 mM KCl, 25 mM Hepes pH 7.4, 33 mM d-Glucose). Some experiments involved a 45-min starvation period in ACSF supplemented with 1 mM MgCl_2_ (ACSF with Mg^2+^) before stimulation to induce amino acids depletion. We controlled that those treatments are compatible with correct neuronal conditions using a calcein viability test (Invitrogen™ Calcein, AM, cell-permeant dye). After a rapid wash with the corresponding medium, DIV 18–21 neurons were incubated at 37 °C for 10 min, with or without drugs in ACSF with (control condition) or without (allowing NMDAR activation) 1 mM MgCl_2_. When phosphorylation of ribosomal S6 was measured, cells were incubated at 37 °C in ACSF with MgCl_2_ for an additional 20-min after the stimulus by adding concentrated MgCl_2_ to reach a concentration of 1 mM (or in CM when indicated). Stimulation in high KCl saline was performed in modified ACSF (high-K^+^ ACSF): 90 mM NaCl, 37.5 mM KCl, 25 mM Hepes–NaOH, 33 mM d-glucose, 2 mM CaCl_2_ and 1 mM MgCl_2_. So called chemical LTP (cLTP) adapted from Lu et al., 2001 [61] was induced in ACSF w/o Mg^2+^ supplemented with glycine (100 µM, Sigma), strychnine (Str, 1 µM, Sigma), and bicuculline (Bic, 20 µM, Sigma). Alternatively, neurons were stimulated by DHPG, a mGluR agonist (50 µM, Tocris), dopamine (50 µM, Sigma) or 50 µM forskolin plus 0.1 µM rolipram (Sigma). BDNF (50 ng/mL, 3.5 nM concentration commonly used to activate TrkB [18, 62, 63], R&D systems) was added for 30–40 min unless otherwise indicated. The drugs listed below were added in CM for 15 min (or indicated time) before the onset of the stimulus then all along the experiment: rapamycin (200 nM, Sigma), ANA-12 (25–50 µM, Tocris), cyclotraxin-B (CTX-B,200 nM, Tocris), d-( −)-2-Amino-5-phosphonopentanoic acid (D-AP5, 50 µM, Tocris), tetrodotoxin (TTX, 500 nM, Tocris), bafilomycin (BafA, 200 nM, Sigma). Amino acids are used at the concentration present in the Neurobasal medium.

### Western blot analysis

Cell extracts (100,000 cells in 4 cm^2^ dishes) were prepared in NuPAGE-LDS sample buffer (Life technologies) for western blot analysis. Proteases (Halt Protease Inhibitor Cocktail, ThermoFisher) and phosphatases inhibitors (phosphatase inhibitor cocktail 2 and 3, Sigma) were added. Extracts were analyzed by SDS-PAGE and immunoblotting. Blots were scanned with ImageQuant™ LAS 4000 (GE Healthcare) and quantified using Image J software [64]. Actin labeling was used as loading control.

### Immunofluorescence

Neurons were washed in PBS and fixed with 4% paraformaldehyde in PBS containing 4% sucrose. They were then washed, permeabilized with 0.1% Triton-X100 in PBS for 3 min and incubated in blocking solution (3% Bovine Serum Albumin (BSA) in PBS) for 30 min at room temperature (RT). Next, they were incubated with primary antibodies diluted in blocking buffer for 1 h at room temperature or overnight at 4 °C. After several rinses, neurons were incubated with the appropriate fluorescent Alexa-conjugated secondary antibodies for 45 min at RT, rinsed extensively in PBS, and mounted with Fluoromount® anti-fading media (Southern Biotech). Due to some prolonged-treatments in ACSF, cell viability was verified by labeling of the nucleus with Hoechst 33,342 (0.01 µg/mL, Thermo Scientific) and the dendrites with anti-MAP2 antibodies.

HeLa cells were fixed with paraformaldehyde 4%, permeabilized and blocked with BSA 5% and saponin 0.2% in PBS for 30 min, and incubated with primary antibodies diluted in blocking buffer for 1 h at room temperature or overnight at 4 °C. After several rinses, cells were incubated with the appropriate fluorescent Alexa-conjugated secondary antibodies for 45 min at RT, rinsed extensively in PBS, and mounted with Fluoromount® media. For HeLa cells, amino acid starvation was performed by incubation for 2 h in Earl’s Balanced Salt Solution (GibCo) and serum starvation by incubation for 12 h in serum-free DMEM.

### Image acquisition and analysis

*Epifluorescence imaging.* Neurons were imaged either using a z-motorized Nikon inverted microscope TE2000E equipped with a 40x (NA 1.3) or a 100x (NA 1.4) objectives, a CCD camera (CoolSNAP ES, Photometrics®), an X-Cite 120Q lamp (Excelitas) and driven with MetaVue™ software (Imaging Research Software); or using a Zeiss microscope (Axio observer Z1) equipped with a 20X objective (NA 0.8), a sCMOS Neo camera (Andor) and an X-cite 120LED lamp (Excelitas) and driven with Slidebook software (3i). In the latter case, each large field image contains 10–20 neurons. Images were analyzed with FIJI software [65].

*P-pS6 imaging quantification.* To specifically quantify the fluorescence intensity of all neurons present in a field, the MAP2 image was thresholded and the resulting mask was transferred to the P-pS6 image to measure the mean fluorescence intensity of the set of neurons. For the differential analysis of somatic and dendritic compartments, the somatic regions were selected by thresholding the P-pS6 full image and the mean fluorescence measured in all neuronal somas present on the image. Then, these regions were removed from the neuronal MAP2 mask to specifically measure the mean P-pS6 fluorescence in dendrites. To study inter-neuronal variability, individual neurons were selected using the segmentation function of the DiAna plugin for FIJI software to obtain P-pS6 mean fluorescence from individual somas. To study the phosphorylation of pS6 along dendrites, line plots made on MAP2 labelled individual dendrites (see Fig. 2) were transferred on P-p6 corresponding image using FIJI software. On all P-pS6 images, background was subtracted using the rolling ball method in FIJI with a diameter of 50 pixels. For some experiments, mCherry-Rheb or CRYII-mCherry-Raptor signals were used to obtain masks corresponding to the cellular surface, that were then transferred to P-pS6 images to measure the mean P-pS6 fluorescence intensity in these cells.

*Quantification of mTOR recruitment to LEs*. mTOR fluorescence was measured in LEs (IN) and outside LEs (OUT) in dendrite images acquired for LAMP1, mTOR, and MAP2 stains by epifluorescence with the 100X objective. At least 10 images per condition were obtained; each one including several dendrites after the removal of the soma. mTOR images were background-subtracted using the rolling ball method in FIJI with a diameter of 75 pixels. Segmentation of LAMP1 signal was done with the Squashh plugin of MosaicSuite for FIJI [66] to provide a mask corresponding to LEs (IN mask). Only structures with a surface above 25 pixels (which corresponds to an average diameter of about 350 nm) were considered. In parallel, the enlarge plugin for FIJI was used on the LAMP1 segmented image to obtain 4 pixel-wide ring-like regions located 6 pixels outside of LE structures and provide a mask corresponding to the neuronal cytoplasm outside of LEs (OUT mask). A mask generated in the MAP2 channel, after applying the mean auto threshold for FIJI, was used to remove out-of-dendrite regions from the IN and OUT masks. Both IN and OUT masks were then transferred to the corresponding background-subtracted mTOR image to measure the mean fluorescence of mTOR in LE structures (mTOR IN signal) and outside surrounding of LE (mTOR OUT signal), respectively. Shown is the ratio (IN/OUT).

*TIRF-SIM microscope.* Lysosomes and mTOR were imaged on a custom-made TIRF-SIM microscope [67]. Super-resolved images were calculated off-line from nine consecutive diffraction-limited images acquired at three different orientations of EW propagation and three different phases each [67] using a phase-optimization technique[68].

### Optogenetics

To photoactivate cells, we used a home-made device. Briefly, 24 white LEDs (#691-0947, RS components) were fixed to thermal interface pads (#752-4982, RS components) arranged on an aluminum plate to fit into wells of a 24-well culture plate. The device comprises three groups of 8 LEDS. Each group is connected to a LED driver (RCD-24-0.70, Recom). The 3 drivers are powered by a 15 V SMPS power supply (#770-3315, RS components) and receive a power width modulation (PWM) signal from an Arduino Uno card (#715-4081, RS components) that enables the user to vary the LED intensity from 0 to 100%. Home-made user interface was written in C++ with Qt-5 libraries. A custom made firmware controlling the Arduino was written in assembly language. The final user can control pulses frequency, width, number and LED power. The device is embedded in a 3D-printed polylactic acid box with holes (5.6 mm in diameter) in front of each LED. An emission filter (475 nm CWL and 50 nm FWHM, Pixelteq, FL, USA) is positioned between the device and the cell culture plate. The optical power density used to photoactivate cells was 2.4 mW/cm^2^, as measured just above the emission filter. Pulse duration was set to 100 ms. Pulses were given in a dark room at a frequency of 0.05 Hz for 5 min for neurons and 0.1 Hz for 5 min for HeLa cells. Cells were either fixed immediately after photoactivation to assess mTOR or CRY2-mCherry-Raptor translocation or kept in the dark for indicated times before fixation to measure pS6 phosphorylation.

### Statistical analyses

Values are given as mean ± S.E.M. Significance of differences between two conditions was calculated with Mann–Whitney U-test or the Student’s t-test when the data were normally distributed. For multiple comparisons, we used ANOVA with Kruskall-Wallis or ANOVA Bartlett’s test (if normal distribution), followed by Dunn’s or Tukey’s post-tests, respectively, using GraphPad Prism version 5.04, GraphPad Software (San Diego, CA, USA). A partial inhibition means that a significant difference is observed both with the stimulated condition and the corresponding control. *p < 0.05, **p < 0.01; ***p < 0.001.

## Results

### NMDA receptors and BDNF trigger mTORC1 activation in hippocampal neurons

To assess the effects of different neuronal stimuli on mTORC1 activity in hippocampal cultures (see schematic in Additional file 1: Figure S1A), we first performed western blot analyses using the phosphorylation of ribosomal S6 protein (pS6) on serines 240 and 244 as a specific readout for mTORC1 activity [69], as pS6 is targeted by p70S6K, a major effector of mTORC1. DIV18 mouse hippocampal cultures were placed in a K^+^-supplemented artificial cerebrospinal fluid (high-K^+^ ACSF) for 5 min to depolarize the plasma membrane and cells were harvested 20 min later to perform western blot analysis [48, 70]. This treatment markedly increased the quantity of phosphorylated pS6 (P-pS6) (Additional file 1: Figure S1B) and was suppressed by the mTOR inhibitor rapamycin (200 nM), indicating an effect on mTORC1 activity. Consistently, the phosphorylation of p70S6K was also increased by high K^+^ treatment (Additional file 1: Figure S1F). K^+^-induced membrane depolarization may act by triggering glutamate release and by relieving the Mg^2+^ block of NMDARs. In agreement with this possibility, removal of Mg^2+.^strongly activated mTORC1, either in ACSF (Additional file 1: Figure S1C) or in ACSF supplemented with NMDAR co-agonist glycine as well as strychnine (a glycine receptor antagonist) and bicuculline (antagonist of GABA-A receptor) (Additional file 1: Figure S1D), a condition known to chemically induce LTP (cLTP) [50, 61]. AP5, a NMDAR antagonist, prevented the increase of P-pS6 induced by these treatments confirming the link between NMDAR activation and mTOR activation (Additional file 1: Figure S1C, D). Of note, Glycine (10–100 µM) had no effect in the presence of Mg^+^ (data not shown), nor strychnine or bicuculline (Additional file 1: Figure S1D). We also investigated the effect of BDNF that is known to activate mTORC1 via the PI3K/Akt/TSC/Rheb pathway and can be released from neurons upon NMDAR stimulation [55, 56, 58]. Addition of BDNF also activated pS6 phosphorylation (Additional file 1: Figure S1E) and ANA12, an inhibitor of the BDNF receptor TrkB, decreased activation of pS6 phosphorylation by high-K^+^ ACSF (Additional file 1: Figure S1G).

**Fig. 1:**
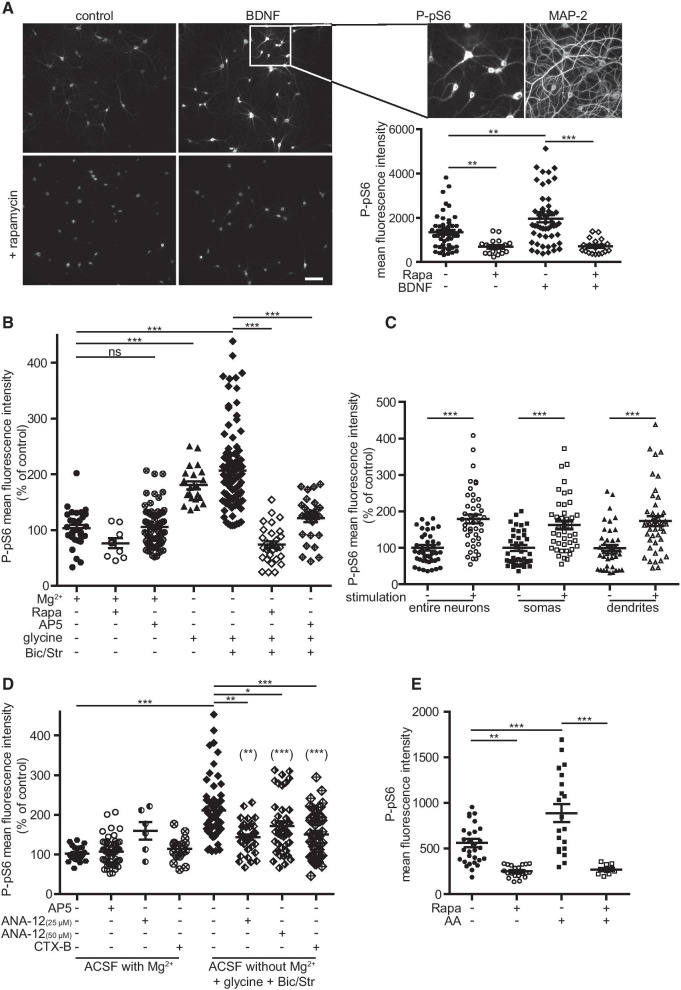
Activation of mTORC1 in neurons by NMDA and BDNF receptors. **A** Epifluorescence images of P-pS6 detected by immunocytochemistry with anti-P-pS6 240/244 antibodies in cultured mouse hippocampal neurons (DIV 18–20) incubated in ACSF with Mg^2+^ for 45 min with BDNF (50 ng/mL), in the presence (Rapa) or the absence of rapamycin (10–25 neurons per large field images). Boxed region is shown at higher magnification with the P-pS6 signal and the corresponding MAP2 images. Shown in the graph is the P-pS6 mean fluorescence in the areas defined as MAP2 positive (BDNF: N = 50 fields from 5 independent experiments; Rapa: N = 20 fields from 2 experiments). Bar, 100 µm. **B** Impact of NMDAR activation on mTORC1 activity. Neurons were treated for 10 min under the indicated conditions and then in ACSF with Mg^2+^ for 20 min before processing for P-pS6 and MAP2 immunolabelling. Removal of Mg^2+^ and addition of glycine (N = 20 fields from 2 independent experiments) or glycine, bicuculline and strychnine (cLTP, N = 100 fields from 10 independent experiments including 3 with rapamycin and 3 with AP5) robustly activated mTORC1. The effect was suppressed by rapamycin and by AP5. **C** P-pS6 mean intensity for the entire neurons, somas or dendrites in control or cLTP conditions (N = 40 fields from 4 independent experiments used in (**B**). Areas tested are similar in both conditions. **D** Neurons were treated as in (**B**) (cLTP conditions) with or without inhibitors of BDNF receptor TrkB, 25–50 µM ANA12 or 200 nM cyclotraxin-B. Both inhibitors partially diminished the effect of NMDAR stimulation (6 independents experiments including 3 with ANA12 25 µM, 4 with ANA12 50 µM or CTX-B, (statistical comparison with ACSF with Mg^2+^ and AP5 are indicated in parentheses). **E** Neurons were starved in ACSF for 45 min, treated for 15 min in ACSF with Mg^2+^ supplemented with amino acids (AA) and processed 15 min later for immunofluorescence. (N = 10–30 large field images per condition from 2 independent experiments)

**Fig. 2:**
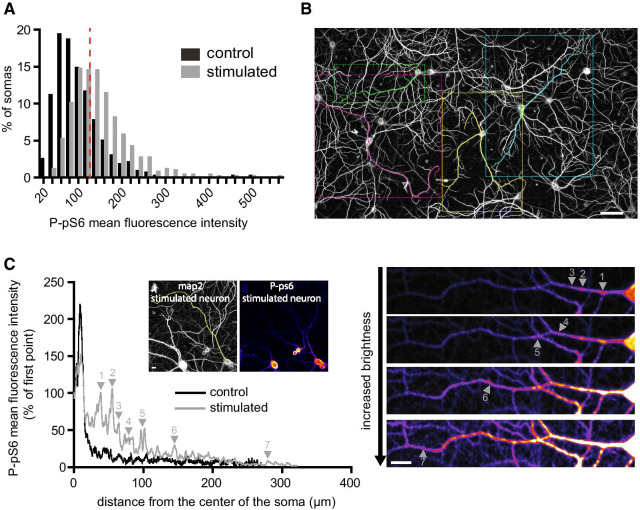
pS6 phosphorylation in dendrites. **A** Variability of neuronal responses to NMDAR stimulation. Shown is the distribution of P-pS6 signal (expressed as mean fluorescence intensity) of individual neuronal somas present in large field images in control or cLTP conditions (N = 439 control somas; N = 517 cLTP somas, 2 independent experiments). The red dotted line represents the 75th percentile of the distribution of the P-pS6 signal measured in control cells. **B** Selection of individual dendrites on a MAP2 epifluorescence image. Bar 50 µm. **C** Presence of active spots far from the soma along dendrites upon NMDAR stimulation. Shown is a representative example of plot line analysis of the P-pS6 signal along dendrites from a control neuron and a cLTP-treated one. Arrows indicate the P-pS6 spots observed in the stimulated dendrites and depicted in the corresponding graph. 20 dendrites from 10 neurons in 2 independent experiments for both conditions have been analyzed. Bar, 20 µm

Next, to define the spatial distribution of mTORC1 activation in neurons, we performed immunofluorescence detection of P-pS6. As described in the Methods section, MAP2 labelling (Figs. 1A, 2B) was used to restrict P-pS6 measurement on the entire neuronal surface present in large field images. We executed all neuronal stimulations in ACSF media devoid of amino acids. Indeed, as in other cell types, neuronal mTORC1 is sensitive to amino acids levels as indicated by immunofluorescence (Fig. 1E) and western blot (Additional file 1: Figure S1H). First, we treated DIV18-21 hippocampal neurons for 40 min with BDNF (50 ng/ml). As expected, BDNF increased the average level of P-pS6 (+ 40.2 ± 6%). This effect was blocked by rapamycin (Fig. 1A), confirming that it involved mTOR activation. Then, to activate NMDARs, we incubated neurons for 10 min in Mg^2+^-free ACSF supplemented with 100 µM glycine, in the absence or in presence (cLTP medium) of strychnine and bicuculline (Fig. 1B). Under those conditions, robust activation of mTORC1 (cLTP: + 109.6 ± 18%) was observed 20 min after the stimulus. Of note, such rapid activation was not observed in the presence of Mg^2+^ or AP5 indicating that it was mediated by NMDARs. Then, we measured P-pS6 immunoreactivity either in somas or in dendrites (Fig. 1C). cLTP treatment induced a robust increase in P-pS6 (at Ser 240/244) in both somas (+ 63 ± 11%) and dendrites (+ 73.5 ± 14%), indicating that, after 30 min, NMDARs-dependent mTORC1 activation was induced throughout the somatodendritic compartment. This AP5-sensitive cLTP-induced mTORC1 activation persisted 90 min after stimulation both in somas and dendrites (Additional file 2: Figure S2). Such somatodendritic distribution of pS6 phosphorylated on Ser 240/244 has already been described in the dentate gyrus after PI3K-dependent mTORC1 activation [71]. Basal levels of mTORC1 activity were also decreased by rapamycin under resting conditions (Fig. 1 and Additional file 2: Figure S2), suggesting that spontaneous neuronal activity may already promote the release of glutamate and BDNF in the medium.

As Ca^2+^ influx through activated NMDARs can trigger BDNF secretion [55, 56] that may provoke Rheb activation leading to mTORC1 stimulation, the contribution of BDNF on mTORC1 activation under NMDARs stimulation was tested. Two inhibitors of the BDNF receptor TrkB, ANA12 and cyclotraxin B, significantly but partially, reduced the effect of NMDAR stimulation on mTORC1 activity (Fig. 1D). Similar results were obtained by western blot quantification of P-pS6 in high-K^+^ medium in the presence of ANA12 (Additional file 1: Figure S1G), confirming that both BDNF and NMDARs contribute to mTORC1 activation in cultured hippocampal neurons.

To explore the variability of neuronal responses regarding mTORC1 activation, we measured P-pS6 signal in individual somas in the large field images analyzed in Fig. 1B. Even though P-pS6 signal intensity was significantly higher in the population of neurons bathed in cLTP medium (p < 0.0001 vs. control, Mann Whitney test), we observed a high heterogeneity in the response of individual neurons. Nevertheless, the distribution of P-pS6 signal intensities was clearly shifted toward higher values and 58% of stimulated neurons displayed a higher P-pS6 intensity than the 75th percentile in control condition (Fig. 2A). We also investigated the distribution of P-pS6 along dendrites (Fig. 2B). In control conditions, P-pS6 was concentrated in proximal dendritic area and gradually decreased along dendrites. Upon NMDAR stimulation, the signal was heterogeneous and several hot spots of fluorescence appeared along dendrites even far away from the soma (Fig. 2C). These results suggest that pS6 is locally regulated by p70S6K and thus by mTORC1 in discrete regions of dendrites. Similar dendritic puncta of phosphorylated (Thr389)-p70S6K have been observed in high-K^+^-stimulated hippocampal neurons [8].

Others pathways have been documented to control mTORC1 activity in neurons. Thus, we probed the effects of dopamine, DHPG, an agonist of type I metabotropic glutamate receptors (mGluR), and forskoline/rolipram that induce an increase in cAMP concentration (often used to induce chemical LTP [10]). None of these drugs had a significant effect on the phosphorylation of pS6 on serine residues 240 and 244 in our conditions (Additional file 1: Figure S1I-J).

### NMDA receptors activation triggers mTORC1 recruitment to late endocytic compartments

A key feature of amino acid-dependent mTORC1 activation in non-neuronal cells is the translocation of mTORC1 to late endocytic compartments (LEs) comprising lysosomes and late endosomes [14]. Translocating mTORC1 to LEs promotes its interaction with and activation by the GTPase Rheb. So far, mTORC1 translocation to LEs has been observed upon addition of amino acids in starved non-neuronal cells. Whether and how mTORC1 translocation is controlled in neurons has not been studied.

**Fig. 3:**
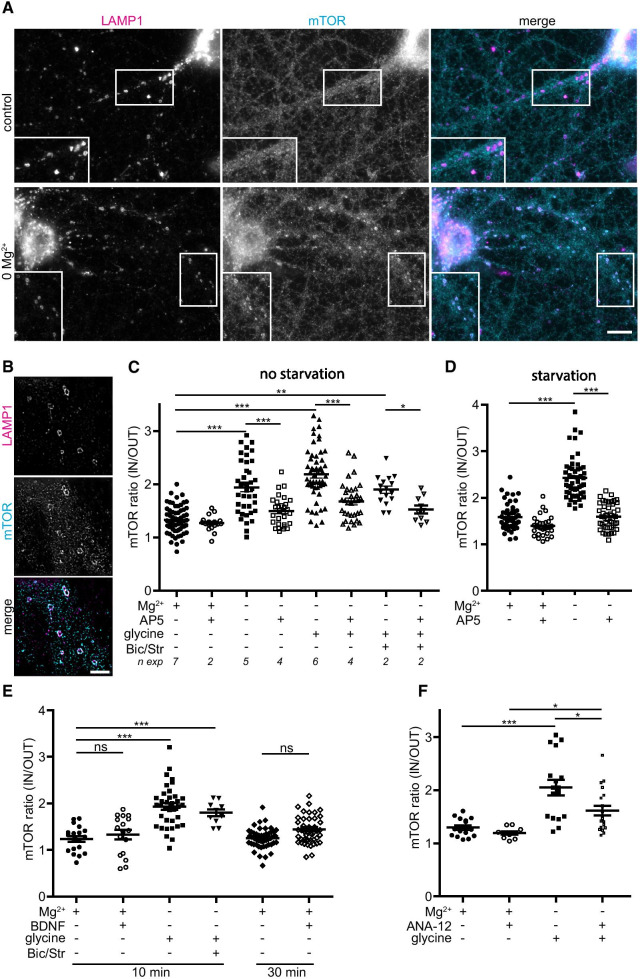
Recruitment of mTORC1 to late endocytic compartments. **A** Changes in the neuronal distribution of mTOR after NMDAR activation. Immunostaining of DIV18 hippocampal neurons for endogenous mTOR and LAMP1, with or without NMDAR stimulation (incubation in Mg^2+^ free ACSF for 15 min). Mg^2+^ removal induced the appearance of clusters of mTOR that co-distribute with LAMP1 positive structures in both soma and dendrites. Bar, 10 µm. **B** Neurons were incubated for 15 min in Mg^2+^-free ACSF, double labelled for mTOR and LAMP1 and imaged with a TIRFM/SIM microscope with a lateral resolution of ~ 100 nm. Structures enriched in mTOR were also positive for LAMP1 confirming that mTOR was recruited on LEs. Bar, 5 µm. **C** Quantification of mTOR recruitment to LEs in dendrites. mTOR fluorescence was measured in LEs (IN) and in the cytosol area surrounding LEs (OUT) in dendrites from 100X epifluorescence images stained for LAMP1, mTOR, and MAP2. Shown is the ratio (IN/OUT) (8–10 images per condition per experiment, *n exp* indicates the number of experiments). Removal of Mg^2+^ induced a rapid AP5-sensitive increase in mTOR recruitment to LEs. Similar results were obtained with Mg^2+^-free ACSF plus glycine or in cLTP. **D** Same as in (**C**) but after a 45 min starvation period in ACSF to reduce possible amino acid-dependency (5 experiments including 3 with AP5). **E** Addition of exogenous BDNF (50 ng/mL in ACSF) does not promote the recruitment of mTOR to LEs after 10 or 30 min (3 and 5 experiments respectively) compared to NMDAR activation (3 experiments for glycine and one for cLTP). (**F**) Contribution of endogenous BDNF to the NMDAR-dependent translocation of mTOR to LEs. Neurons were treated as in (**C**) in ACSF or in Mg^2+^-free ACSF plus glycine. The TrkB inhibitors ANA12 (25 µM) reduced the recruitment of mTOR to LEs (2 experiments)

We examined the subcellular localization of mTOR kinase in cultured hippocampal neurons by immunocytochemistry. In these experiments, LAMP1 was used as a marker of LEs. LAMP1 positive structures of various sizes were seen in both soma and dendrites (Fig. 3A). Under basal conditions, mTOR displayed a diffuse distribution in the cytoplasm. However, upon Mg^2+^ removal, mTOR was clearly enriched at the membrane of LAMP1 positive compartments both in dendrites and cell soma (Fig. 3A). In many instances, mTOR and LAMP1 positive structures displayed a donut-like shape strongly suggesting that these structures were LEs. We used a TIRFM-SIM microscope with a lateral resolution of ~ 100 nm and an axial confinement of ~ 100 nm to further address the identity of the compartment to which mTOR was targeted [67]. As shown in Fig. 3B, in neurons treated with Mg^2+^-free ACSF, most LAMP1 positive LEs were decorated with anti-mTOR antibodies; and almost all structures with mTOR labelling above background level were LAMP1 positive. We therefore conclude that the structures onto which mTOR is recruited in neurons upon stimulation are LEs.

We compared the effect of different treatments on mTOR recruitment on LEs. Epifluorescence LAMP1 masks (selecting structures above 300 nm in diameter) were transferred to mTOR images to assess dendritic mTOR labelling in LAMP1-positive structures (IN) and surrounding LAMP1-negative regions (OUT). In control conditions, the mean IN/OUT ratio was 1.33 ± 0.03 (n = 59 images) indicating a low amount of mTOR kinase on these structures. As shown in Fig. 3C, incubating cells in Mg^2+^-free ACSF for 2 (data not shown)-10 min induced a significant increase in the concentration of mTOR in LAMP1 positive regions (mean IN/OUT ratio = 1.94 ± 0.08, n = 39 images). mTORC1 translocation to LEs was even more robust in Mg^2+^-free ACSF supplemented with glycine (mean IN/OUT ratio = 2.18 ± 0.07, n = 55 images) with or without bicuculline and strychnine. This effect was suppressed by AP5 (Fig. 3C) or MK801 (not shown), indicating that NMDAR activation was responsible for mTOR recruitment at the membrane of LEs. When the same experiments were performed after a 45 min starvation period in ACSF, NMDAR-dependent mTOR translocation was still observed indicating that it can occur in the absence of external amino acids (Fig. 3D). However, when cells were treated with BDNF for 10 or 30 min, mTOR was not found to be enriched on LEs compared to control cells (Fig. 3E). To explore a potential effect of endogenous BDNF secretion through activated NMDAR on mTOR translocation, we treated neurons with Mg^2+^-free solution plus glycine in the presence or absence of the TrkB inhibitors ANA12 (Fig. 3F and Additional file 3: Figure S3A) or cyclotraxin-B (Additional file 3: Figure S3B). Surprisingly, the effect of NMDAR stimulation on mTOR translocation was reduced, albeit not suppressed, by TrkB inhibitors (Fig. 3F) even in starvation conditions (Additional file 3: Figure S3A, B). The v-ATPase inhibitor bafilomycin A (BafA) has also been shown to interfere with this translocation process [33, 72]. We therefore probed the effect of this drug on NMDAR-induced mTOR translocation. As shown in Additional file 3: Figure S3C, mTOR translocation observed upon Mg^2+^ removal was suppressed by BafA under starvation conditions. These data suggest that NMDARs trigger mTOR translocation by a mechanism similar to the effect of intracellular amino acids described in non-neuronal cells.

**Fig. 4:**
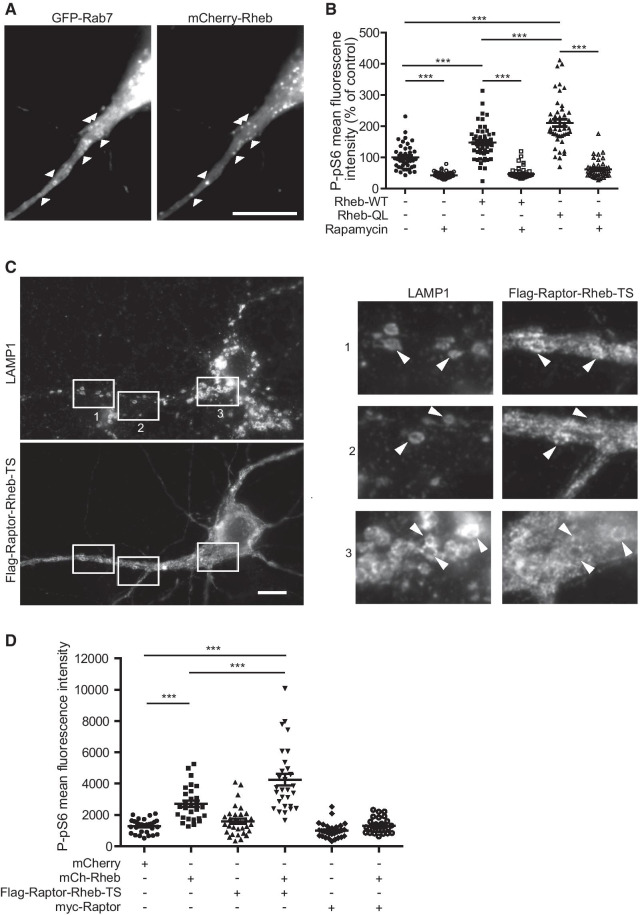
Synergistic activation of mTORC1 by Rheb and LE-associated mTOR. **A** Association of Rheb with LEs. DIV 13 hippocampal neurons were transfected with mCherry-Rheb and GFP-Rab7 and fixed three days later. Shown is an epifluorescence image of a dendritic region. A majority of Rheb positive structures are Rab7 positive (examples are depicted with arrowheads). Bar, 10 µm. **B** Overexpression of Rheb in neurons activates mTORC1 pathway. DIV 13 neurons were transfected with WT or Q64L mCherry-Rheb and 4 days’ later immunofluorescence for P-pS6 was performed with or without 3 h pre-incubation with 100 nM rapamycin. Shown is P-pS6 intensity of transfected neurons expressed as percentage of the mean fluorescence measured in control neurons (transfected with empty mCherry vector) from two independent experiments with 40–50 neurons per conditions. **C** Epifluorescence images of hippocampal neurons transfected with Flag-Raptor-Rheb15-TS and labeled with anti-LAMP1 and anti-Flag antibodies. Regions boxed in C are shown at higher magnification on the right panel. Most of LAMP1-positive structures are also positive for Flag-Raptor-Rheb15-TS. Bar, 10 µm. **D** DIV14 neurons were transfected with mCherry or mCherry-Rheb, Flag-Raptor-Rheb15-TS or myc-Raptor as indicated and 4 days later labeled with anti-P-pS6 and anti-Flag or anti-myc antibodies. Neurons expressing the constructs were imaged for P-pS6 intensity. Flag-Raptor-Rheb15-TS alone had no effect on P-pS6 levels but increased mTORC1 activity when combined with Rheb overexpression (N = 30 images from two independent experiments)

The translocation of mTORC1 to LEs would not lead to mTOR activation if Rheb would not be associated with LEs in neurons. We thus addressed the intracellular distribution of Rheb in dendrites of cultured hippocampal neurons. Several commercial anti-Rheb antibodies were tested but did not provide convincing data. We therefore analyzed the distribution of transfected mCherry-tagged Rheb. We found that mCherry-Rheb was predominantly associated with LAMP1 or GFP-Rab7 positive compartments either in resting conditions or after a 10 min stimulation in Mg^2+^-free ACSF supplemented with glycine (Fig. 4A). We also observed that overexpression of Rheb-WT or its constitutively active mutant RhebQ64L induced, by itself, an increase in the phosphorylation of pS6 on residues 240/244 (Fig. 4B), in a dose-dependent manner (not shown), indicating that Rheb GTPase may be a limiting factor in mTORC1 activation in neurons. Similar results were obtained when measuring pS6 phosphorylation on serine 235/236 in hippocampal neurons after Rheb overexpression [50] (data not shown).

### Functional impact of mTORC1 translocation to late endosomes and lysosomes

We next examined whether there is a functional link between the induction of mTOR translocation to LEs and mTORC1 activation. To address such a link, we designed experiments aimed at measuring the functional impact of mTOR targeting to LEs. First, the mTORC1 component Raptor was fused to the last 15 residues of Rheb coding for its intracellular targeting signal (TS) that is sufficient to address Rheb to LEs. The resulting construct (Raptor-Rheb15-TS), was previously shown to be localized on LEs in non-neuronal cells [35]. In agreement with this report, we found that Raptor-Rheb15-TS was co-localized with LAMP1 in transfected neurons, indicating that it was predominantly addressed to LEs (Fig. 4C) and could be used to target mTOR to LEs*.* Overexpressing Raptor-Rheb15-TS did not increase pS6 phosphorylation by itself (Fig. 4D). This lack of effect of Raptor-Rheb15-TS may reflect low levels of active Rheb on LEs under the conditions used. Indeed, co-expression of mCherry-Rheb, in conditions leading to a moderate increase in pS6 phosphorylation, and Raptor-Rheb15-TS, synergistically increased mTORC1 activity (Fig. 4D). The effect was dependent on LE targeting of Raptor because it was not observed with Myc-tagged Raptor lacking the LE-targeting motif (Fig. 4D). These results suggest that mTORC1 association with LEs promotes mTOR activity when active Rheb level is not limiting. Surprisingly, co-expression of mCherry-Rheb with Myc-Raptor lacking the LE-targeting motif prevented the activating effect of overexpressing Rheb alone on mTORC1 (Fig. 4D). This could be due to a sequestration of mTOR away from the lysosome and hence from Rheb by excess of soluble Raptor.

**Fig. 5:**
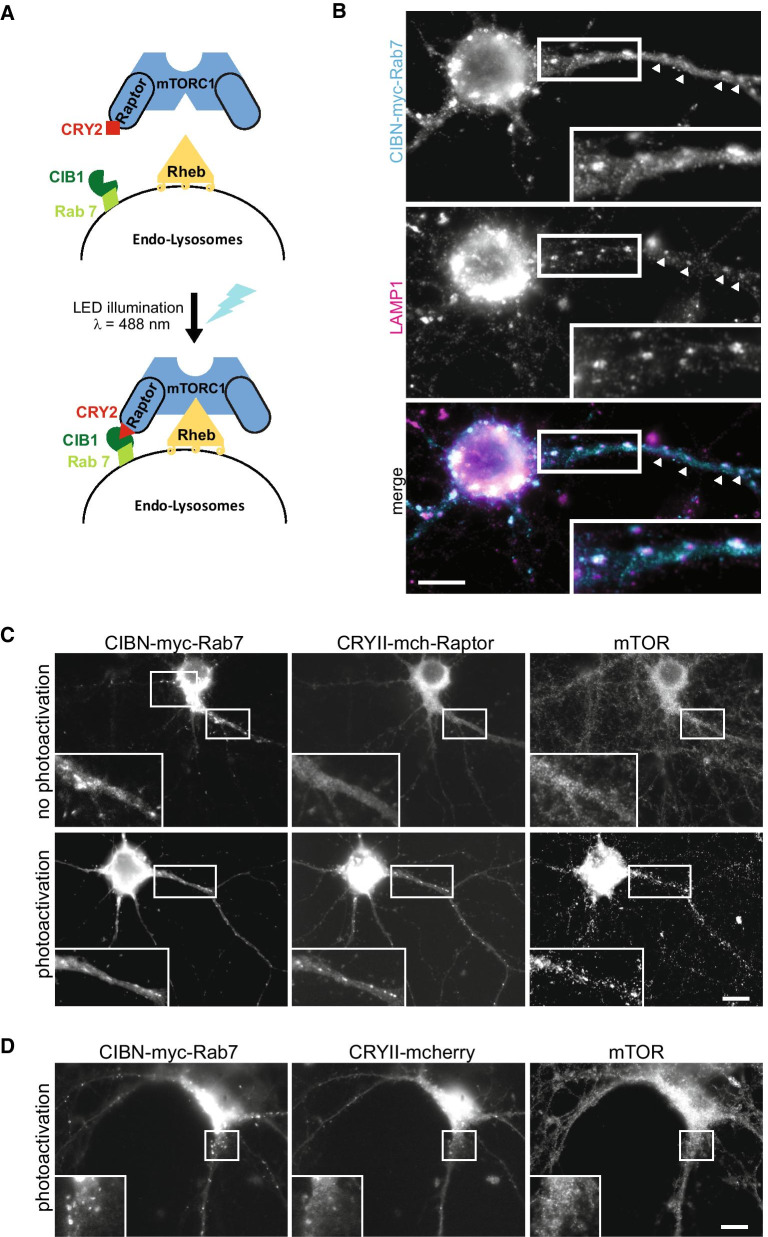
Targeting mTOR to late endocytic compartments with photosensitive dimerizers. **A** Schematic representation of the optogenetic modules used to recruit mTOR on LEs. **B** Epifluorescence images illustrating the intracellular distribution of CIBN-myc-Rab7 on LAMP1 positive structures. Hippocampal neurons were transfected with CIBN-myc-Rab7 and immunolabeled for LAMP1 and myc. Bar, 10 µm. **C** DIV14 neurons were transfected with CIBN-myc-Rab7 and CRYII-mCherry-Raptor; two days later, cells were either kept in the dark (top images) or photoactivated for 5 min (15 × 100 ms-pulses of blue light at 2.42 mW/cm^2^; bottom images) before being processed for mTOR immunofluorescence. Upon photoactivation CRYII-mCherry-Raptor and endogenous mTOR are targeted to CIBN-myc-Rab7 positive structures. Boxed regions are shown at higher magnification **D** When CRYII-mCherry-Raptor was replaced with CRYII-mCherry, photoactivation induced the clustering of CRYII-mCherry on LEs but mTOR remained in the cytosol, as expected. Bar, 10 µm

To circumvent possible drawbacks due to long-term expression of Raptor-Rheb15-TS, we addressed the same question by an optogenetic approach. We used light-sensitive dimerizers to create constructs with the unique ability to control, in a spatio-temporal way, mTORC1 targeting to LEs upon blue light delivery. To do so, mCherry-Raptor was fused to cryptochrome 2 (CRY2) and the LE marker Rab7 was fused to CIBN. Upon illumination, CRY2 undergoes a conformational change that allows its transient association with CIBN [73] (Fig. 5A). CIBN-myc-Rab7 was correctly targeted to LEs in HeLa cells (Additional file 4: Figure S4A) and in neurons (Fig. 5B). Upon exposure to brief pulses of blue light, CRY2-mCherry-Raptor was efficiently recruited to LEs in both cell types (Additional file 4: Figure S4B, Fig. 5C) indicating efficient binding to CIBN-Rab7. In HeLa cells, this optogenetic tool was able to provoke the targeting of both CRY2-mCherry-Raptor (Additional file 4: Figure S4B) and mTOR (Additional file 4: Figure S4C) to LEs, reversibly, within a few minutes, upon light delivery (Additional file 4: Figure S4D). In addition, this recruitment led to an increase in mTORC1 activity 30 min later (Additional file 4: Figure S4D), an effect blocked by rapamycin (Additional file 4: Figure S4E). We also noticed that, after photoactivation, CIBN-myc-Rab7/CRY2-mCherry-Raptor/mTOR enriched structures have the tendency to disperse in the cell.

**Fig. 6:**
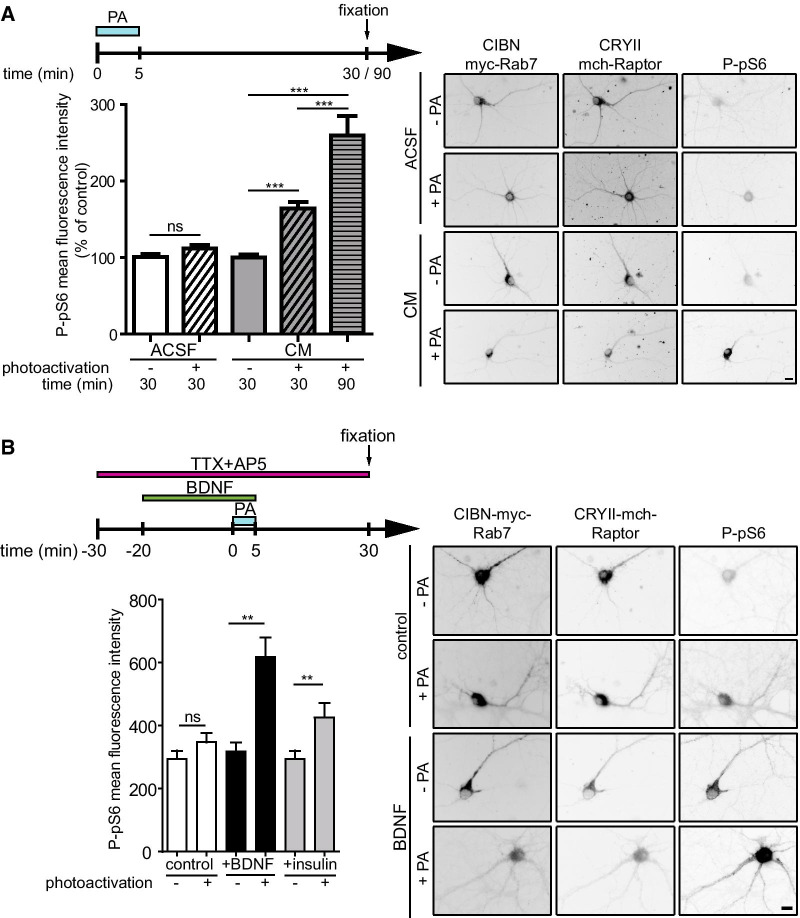
Optogenetic activation of mTORC1 in neurons. Hippocampal neurons were transfected and photoactivated as in Fig. 5. **A** Neurons were bathed either in ACSF or in conditioned medium (CM), and processed for P-pS6 immunofluorescence 25 min or 85 min after the end of photoactivation (PA), as indicated. Photoactivation increased P-pS6 in CM (N = 45 neurons per condition from 3 independent experiments) but not in ACSF (N = 30 neurons per condition from 2 independent experiments). Images of representative neurons for each condition are shown on the right. **B** Synergistic activation of mTORC1 by photoactivation and BDNF or insulin. Neurons were bathed in CM containing 500 nM TTX and 50 µM AP5 to reduce basal levels of mTORC1 activity. Under these conditions, without photoactivation, BDNF or insulin had no significant effect on P-pS6 by themselves. Photoactivation triggered an increase in P-pS6 when it was combined with BDNF or insulin treatment (N = 60 images per condition from two independent experiments in both conditions). Images of representative neurons for each condition are shown on the right. Bar, 10 µm

In CIBN-myc-Rab7-expressing neurons, upon light exposure, mTOR was also efficiently recruited to LEs in cells expressing CRY2-mCherry-Raptor (Fig. 5C) but not in those expressing CRY2-mCherry (Fig. 5D). We then measured the effects of this recruitment on mTORC1 activity. When transfected cells were bathed in ACSF, photoactivation did not induce significant increase in pS6 phosphorylation even after a 25-min delay (Fig. 6A). However, when neurons were bathed in conditioned medium (CM), P-pS6 level increased upon photoactivation (Fig. 6A). This increase observed 30 min after illumination was maintained 90 min later (Fig. 6A). At that point, mTORC1 was already dissociated from LEs due to the reversibility of the conformational change of CRY2 leading to its dissociation from CIBN within 10–15 min (Additional file 4: Figure S4D). The difference observed between ACSF and CM could be due to the presence in CM of amino acids or growth factors that power the PI3K/Akt pathway and thus the amount of active Rheb. We thus determined if treatments known to activate Rheb, such as BDNF and insulin, could potentiate the effect of photoactivation on mTOR activity. To do so, we selected conditions under which BDNF or insulin had little effect on P-pS6 levels by themselves. A 20-min treatment with BDNF or insulin, in the presence of TTX and AP5 that blocked spontaneous neuronal activity, had no effect on pS6 phosphorylation (Fig. 6B). Interestingly, in the presence of TTX and AP5, photoactivation had also little effect on P-pS6 even in CM. In contrast, combined with BDNF or insulin, photoactivation strongly activated mTORC1 (Fig. 6B). Rapamycin inhibited this effect (data not shown). Similar experiments done in HeLa cells (Additional file 4: Figure S4F), also showed that light-induced mTORC1 activation occurred only in medium enriched in serum (Additional file 4: Figure S4F). In this experiment, deficiency in amino acids attenuated light-induced mTORC1 activation but did not prevent it. Overall, these data indicate that mTORC1 translocation to LEs promote mTOR activity only when the amounts of active Rheb are not limiting.

## Discussion

### Synergistic roles of NMDAR activation and BDNF in mTOR localization and activation

Despite its known function as a ubiquitous cellular integrator of both nutritional states and environmental conditions to adapt the balance of anabolism and catabolism, mTORC1 has specific functions in neurons that are not fully explored at the molecular level and dysregulations of these functions have been linked to several neuronal pathologies [14, 23, 53]. In this paper, we enlighten how NMDAR activation promotes protein synthesis at the synapse during LTP and provide an explanation for the combined action of NMDARs and BDNF on mTORC1 in hippocampal neurons. Previous work has shown that NMDAR can elicit postsynaptic BDNF release and autocrine TrkB activation, which may in turn stimulates the PI3K/Akt/Rheb/mTORC1 pathway, causing changes in mRNA translation and the synaptic proteome [54–56]. Consistently, we show that acute NMDAR stimulation leads to mTORC1 activation and this is partially inhibited by TrkB inhibitors, suggesting a contribution of BDNF release and TrkB activation. However, BDNF release cannot be the only mechanism responsible for the observed NMDAR-dependent mTORC1 activation. Indeed, BDNF hardly increased mTORC1 activity in the presence of TTX and the NMDAR inhibitor AP5 (Fig. 6). Consistently, it was shown that BDNF increased the synthesis of the synaptic protein Arc in a NMDAR-dependent manner [74]. We show that opening NMDARs triggers the translocation of mTORC1 to LEs thereby promoting the binding of mTOR to its activator Rheb. Hence, NMDARs, by eliciting mTORC1 translocation, and BDNF, by activating the PI3K/Akt/TSC/Rheb pathway, power the two arms of the mTORC1 activation mechanism (schematic in Additional file 1: Figure S1A).

So far, the mechanism of mTORC1 translocation to LEs has been highlighted in the context of amino acid sensing. Note that mTORC1 recruitment induced by this NMDAR-dependent pathway occurs in glucose rich-medium devoid of amino acids and growth factors and therefore did not rely on amino acids influx. However, we found that neuronal recruitment of mTORC1 to LEs was inhibited by bafilomycin, suggesting that it involves v-ATPase [33]. Therefore, at least part of the amino acid-sensitive mechanism is used by the NMDAR-dependent pathway disclosed in this study. How NMDAR or TrkB activation impinge on the amino acid-sensitive machinery remains to be elucidated. Rag GTPases are also localized to lysosomes in neurons of the mature mouse brain [75]. Future work will determine if they mediate NMDAR-dependent mTORC1 recruitment to LEs.

While addition of exogenous BDNF was able to slightly activate mTORC1 in cultured neurons, it did not have any significant effect on mTORC1 localization, suggesting that there is enough mTORC1 on LEs under resting conditions to respond to an increase in GTP-bound Rheb. On the other hand, both NMDAR-mediated activation of mTORC1 and NMDAR-mediated translocation of mTORC1 to LEs were partly dependent on TrkB activation. This may reflect an effect of Rheb on mTORC1 recruitment onto LEs [24]. Rheb via its Cter-farnesylation is associated with LEs by an unknown targeting mechanism [76]. We observed that Rheb is associated with LEs in hippocampal neurons and recent structural studies suggest a central position of activated Rheb in the complex associating Rag and mTORC1 to the lysosome surface [24, 31, 32] and show that Rheb binds to mTOR at a site remote from its kinase active site resulting in a conformational change that favors mTOR activity [26]. This model is consistent with the simultaneous interaction of mTORC1 with both the Rag GTPases and Rheb, leaving the catalytic site facing the cytosol to allow mTORC1 to fulfill its function as a kinase [24, 32]. We postulate that Rheb could contribute to mTORC1 recruitment onto LEs and that BDNF, via Rheb activation, may also promote or stabilize this recruitment.

### Controlling the recruitment of mTOR onto lysosomes emphasizes its role in mTOR activation

We also report on an optogenetic module endowed with the ability to target mTOR to LEs. When the activity of Rheb is not limiting, this module provides spatial and temporal control over mTORC1 activity. Compared to approaches such as Raptor-Rheb15-TS overexpression, the optogenetic module offers the opportunity to trigger mTOR activity acutely and reversibly, or to tune mTORC1 response with different patterns of photoactivation. This might also be done in vivo without any potential drawback associated with chronic mTOR overactivation. Finally, one could photoactivate only a selected region of a cell to address the effect of locally activating mTOR or to analyze mTORC1 effects without the difficulties associated with between-cells comparisons. Using this module, we found that light-induced mTORC1 translocation to LEs increased its activity in neurons, but only in the presence of enriched-media or upon simultaneous BDNF or insulin treatment, two conditions known to induce Rheb activation. This effect is consistent with the one of Raptor-Rheb15-TS. The amount of GTP-bound Rheb GTPase thus seems to be a limiting factor in the process of mTOR activation. Indeed, we observed that Rheb overexpression was able to increase mTORC1 activity. An effect on spine morphology was also described for RhebQ64L overexpression combined with glycine treatment [50]. Of note, Rheb was originally identified as a gene upregulated upon neuronal activity in rat brain [77]. Interestingly, mTOR signaling becomes less sensitive to nutrients in Rheb over-expressing cells [76]. Light-induced mTORC1 recruitment was reversible within less than 20 min while the effect of photoactivation on pS6 phosphorylation was stable for at least 90 min, indicating that P-pS6 slowly returns to the unphosphorylated state or that mTORC1 remains active even after dissociation from the LE membrane. The latter possibility would be consistent with data obtained in cell lines suggesting that mTORC1 is transiently recruited to LEs, yet durably activated after it detaches from these organelles [70]. However, a recent work suggested that mTOR is active only when localized at the surface of LEs and proposed that a spatial cycling of mTOR between the surface of LEs and the cytosol in the presence of amino acids would constitute a mechanism to attenuate and maintain a physiological level of mTOR activity [78]. Thus, the dynamics of mTORC1 recruitment at the surface of LEs and activation are not clearly established and will need further studies.

So, we found that photoactivation did not promote mTORC1 activity in ACSF whereas mTORC1 was translocated to LEs under these conditions. Hence, mTORC1 recruitment is not sufficient to activate mTOR when the PI3K/Akt/TSC/Rheb pathway is not activated. Therefore, the fact that NMDAR activation in ACSF powers mTOR activity demonstrates that it provokes both mTORC1 translocation to LEs and Rheb activation, most likely via induction of BDNF release. Such requirement of both NMDAR activation and BDNF release on mTORC1 activation has been documented for local dendritic spine enlargement [50, 55]. Accordingly, the fast antidepressant effect of ketamine was suggested to result from a long-lasting decrease in inhibitory neurotransmission causing an increase in excitatory neurotransmission leading in turn to improved synaptogenesis via a mechanism involving activity-dependent release of BDNF and mTORC1 signaling including its downstream effectors 4E-PB1 and 4E-PB2 [79–81]. Recently, a Sestrin modulator, NV-5138, has been shown to produce a similar rapid and long-lasting antidepressant effect also by improving mTORC1 activation and synaptic plasticity in a BDNF-dependent manner [82]. As NV-5138 prevents Sestrin interaction with GATOR2 [83], it could act by favoring mTORC1 recruitment to LEs [38], thus bypassing NMDAR activation.

Altogether, our data indicate that dendritic LEs constitute a signaling platform integrating synaptic inputs and tuning mTORC1 activity in neurons. Beyond the role of LEs in substrate degradation, there is clear evidence that LEs have a crucial function also in neurons as a platform regulating metabolic signaling, nutrient sensing, and stress, related to their mobility and cellular localization [23, 53]. LE positioning and mTOR signaling are linked. Nutrient-dependent-kinesin-transport of LEs to the cell periphery promotes mTORC1 activation where growth factor signaling occurs [84]. In neurons, LEs are present not only in the cell soma but also in dendrites and near dendritic spines and are mobilized upon neuronal activity; then they are well positioned to orchestrate the balance for anabolic-catabolic processes involved in synaptic plasticity [85, 86]. The precise characterization of the nature of this dendritic endo-lysosomal pool and the regulation of its intracellular distribution coupled with mTOR activation upon synaptic activity remains to be defined. Knowledge of new mechanisms contributing to mTOR pathway modulation in brain may hold promising therapeutic strategies to improve certain disease states.

## Supplementary Information

**Additional file 1: Figure S1.** mTORC1 activity in cultured hippocampal neurons. A) Experimental model to study postsynaptic mTORC1 activation upon NMDAR stimulation and autocrine BDNF release causing TrkB activation. AP5 is an NMDAR antagonist. ANA12 and cyclotraxin-B (CTX-B) are TrkB inhibitors. B-D) DIV 18 hippocampal cultures were incubated in ACSF with Mg^2+^ (control) and treated or not for 5 min with high-K^+^ ACSF (in B) or in Mg^2+^-free ACSF to activate NMDARs (in C) or in Mg^2+^-free ACSF in the presence of a combination of glycine, bicuculline and strychnine (in D), 20 min later, cells were harvested and processed for western blot analyses for P-pS6. Actin was used as loading control. Representative blots from 3–7 independent experiments per conditions are shown. Showing increased levels of P-pS6 (+ 111 ± 18% for high K^+^, n = 7; + 95 ± 13% for cLTP, n = 7). The effect on P-pS6 was suppressed by AP5 indicating that it was dependent on NMDARs and by rapamycin indicating increased activity of mTORC1. E) Incubation with BDNF (50 ng/mL) for 45 min in ACSF also increased the level of P-pS6 to a lower extent (+ 69 ± 20%, n = 4). F) Increased phosphorylation of Thr389-p70S6K in neurons stimulated in high-K^+^ saline as in (B). Shown is one blot out of two independent experiments. G) Inhibiting TrkB receptors with ANA12 reduced pS6 phosphorylation in neurons stimulated with high K^+^-ACSF as in (B). Shown is a representative western blot out of 3 independent experiments. H) Addition of either a cocktail of 20 amino acids (AA) or of only Leu, Gln and Arg for 15 min is also able to increase P-pS6 in neurons, as measured 15 min later by western blot (a representative western blot out of 2 independent experiments). I) DIV20 hippocampal neurons were treated for 10 min in ACSF containing TTX and AP5, with forskolin and rolipram or with dopamine with or without rapamycin (added 2 h before stimulation) and processed later for immunofluorescence to assess P-pS6 levels. Treatments aimed at specifically increasing PKA had little effect on mTORC1 activity. J) A 15-min treatment with DHPG, an agonist of group I metabotropic glutamate receptors, in ACSF had no significant effect on P-pS6 levels. (N = 20 images in I and J from 2 independent experiments).

**Additional file 2: Figure S2.** Increased pS6 phosphorylation 90 min after NMDAR stimulation in soma and proximal dendrites. Neurons were treated in cLTP medium as in Fig. 1B with or without AP5 or rapamycin as indicated, then incubated again in conditioned medium (CM) for 80 min before P-pS6 and MAP2 immunofluorescence. Note that over such extended-duration treatments, ACSF was replaced by CM to preserve neuron health. Individual neurons were imaged and P-pS6 quantified in soma or proximal dendrites (mean length 75 µM) for 40–60 neurons from 3 experiments.

**Additional file 3: Figure S3.** TrkB inhibitors ANA12 or cyclotraxin-B reduced the recruitment of mTOR to LEs (IN/OUT ratio, see Fig. 3) induced by NMDAR- activation even in starvation conditions. Neurons were treated as in Fig. 3D in ACSF plus Mg^2+^ or in Mg^2+^-free ACSF, as indicated A) Effect of ANA12 (25 µM) on mTOR IN/OUT ratio from 3 independent experiments. B) Effect of Cyclotraxin-B (CtxB) on mTOR IN/OUT ratio from 2 independent experiments. In stimulated neurons treated with the TrkB inhibitors, the IN/OUT mTOR ratio stays significantly higher than the one observed in non-stimulated neurons. C) Bafilomycin-A (BafA 200 nM) prevented the translocation of mTOR to LEs triggered by Mg^2+^ removal as in Fig. 3D (N = 20 images per condition from 2 independent experiments).

**Additional file 4: Figure S4.** Set up of optogenetic activation of mTORC1 in HeLa cells. A) CIBN-Rab7 was correctly targeted to LEs in HeLa cells transfected with CIBN-myc-Rab7 and immunolabeled for LAMP1 and myc tag. B) Photoactivation was able to target CRY2-mCherry-Raptor to LEs. HeLa cells were transfected with CIBN-myc-Rab7 and CRYII-mCherry-Raptor; 24 h later cells were either kept in the dark (left panel) or photoactivated for 5 min (30 × 100 ms-pulses of blue light given at 0.1 Hz at 2.42 mW/cm^2^) (right panel) before being processed for immunofluorescence. C) Photoactivation (as in B) was able to target mTOR with CRY2-mCherry-Raptor to LEs. D) Left, clustering of CRY2-mCherry-Raptor on LEs induced by photoactivation reverses within a few min. Shown is an index of raptor clustering (intensity (max/min)/mean) per pixel obtained on raptor surface area). Right, the same treatment induces effective pS6 phosphorylation only 30 min after it was given. Experiments were performed as in (B) with immunofluorescence done at different times after illumination (PA) as indicated. Cells were incubated in culture medium after illumination (N = 90 images per condition from three independent experiments). E) Rapamycin blocks photoactivation-induced P-pS6 increase (N = 90 images per condition from three independent experiments). F) The effect of photoactivation-induced mTOR recruitment to LEs on mTOR activity depends on FCS but only partially relies on amino acids. Cells were fed ( +) or starved (-) with serum (FCS) or amino acids (AA) for the time indicated in the diagram. In AA-starved cells, photoactivation induces an increase in P-pS6, although significantly reduced in comparison to cells fed with both AA and FCS. FCS starvation suppresses mTORC1 activity even after photoactivation (N = 60 images per condition from two independent experiments).

## Abbreviations

ACSF: Artificial cerebrospinal fluid
BDNF: Brain-derived neurotrophic factor
LE: Late endocytic compartment
L-LTP: Late phase long-term potentiation
mTORC1: Mechanistic target of rapamycin complex 1
NMDAR: N-methyl-D-aspartate receptor
PI3K: Phosphatidylinositol 3-kinase
P-p70S6K: Phosphorylated (Thr389)-p70S6 kinase
P-pS6: Phosphorylated S6 ribosomal protein on serines 240 and 244
Rheb: Ras homolog enriched in brain
TrkB receptor: Tropomyosin receptor kinase B
