# Supplementary figures and images for: A role for BDNF- and NMDAR-induced lysosomal recruitment of mTORC1 in the regulation of neuronal mTORC1 activity
Source: Mol Brain. 2021 Jul 12;14:112. doi: 10.1186/s13041-021-00820-8 (PMC8273036; doi:10.1186/s13041-021-00820-8)

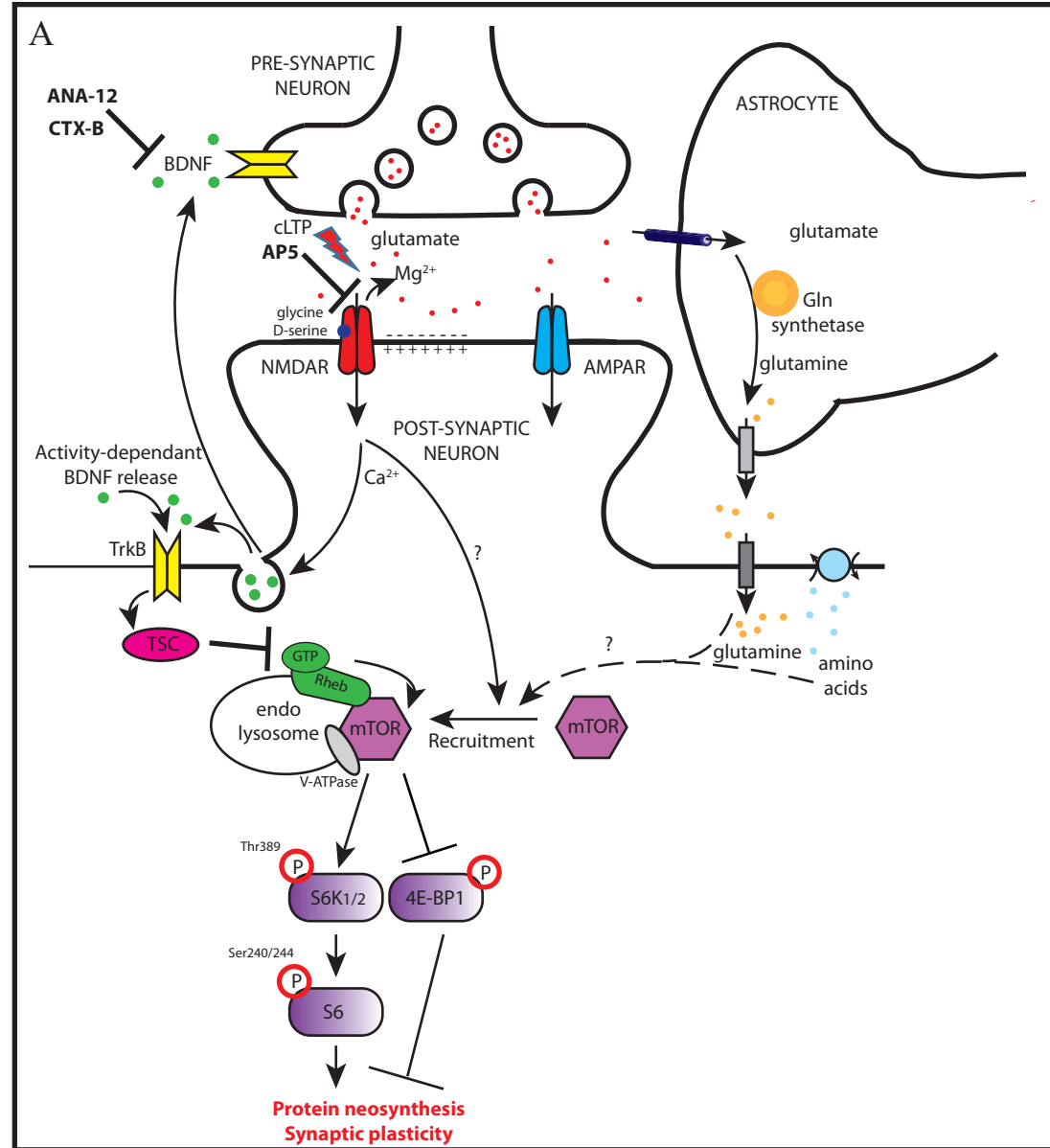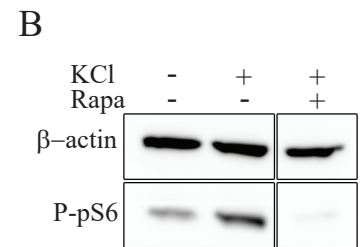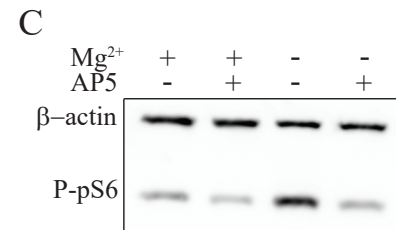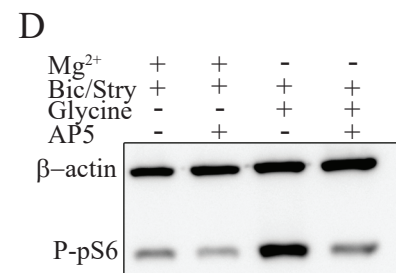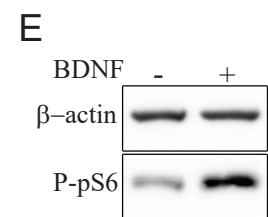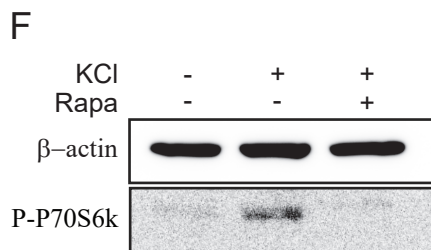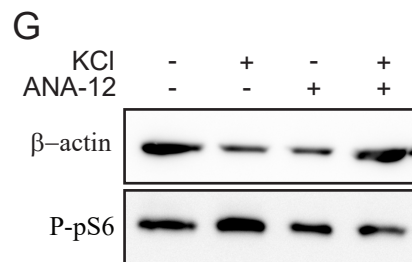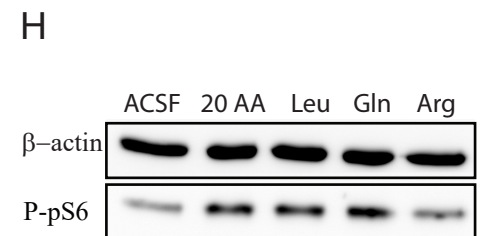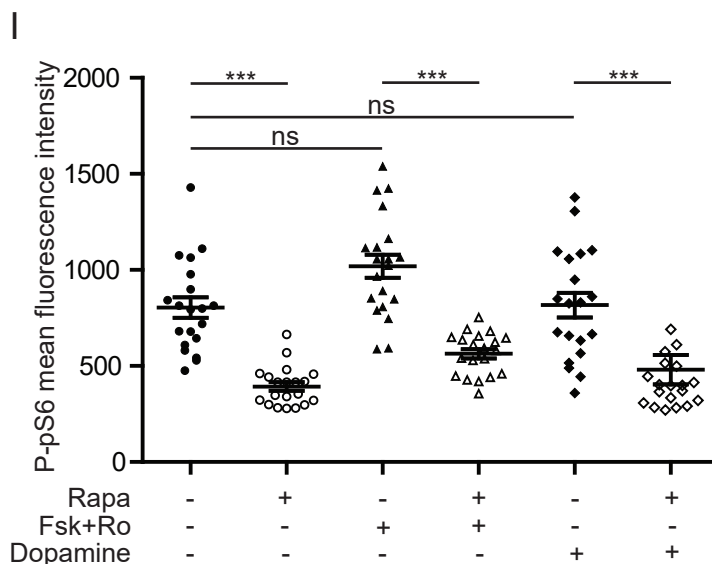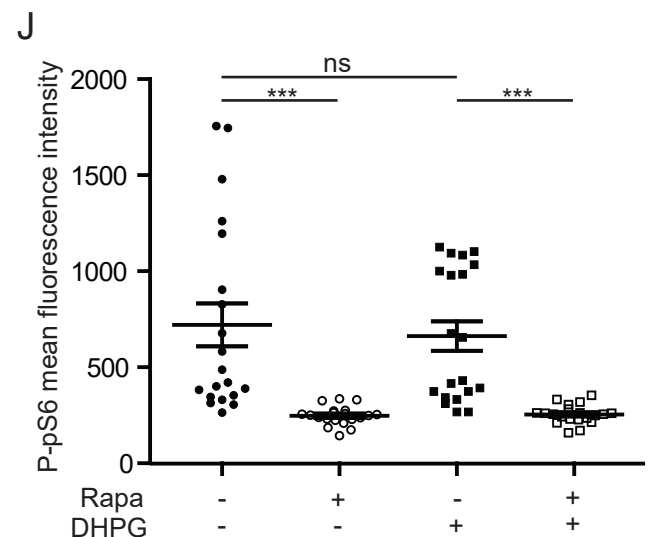

Additional figure 1

Supplement: Supplementary file 1 — Additional file 1: Figure S1. mTORC1 activity in cultured hippocampal neurons. A) Experimental model to study postsynaptic mTORC1 activation upon NMDAR stimulation and autocrine BDNF release causing TrkB activation. AP5 is an NMDAR antagonist. ANA12 and cyclotraxin-B (CTX-B) are TrkB inhibitors. B-D) DIV 18 hippocampal cultures were incubated in ACSF with Mg2+ (control) and treated or not for 5 min with high-K+ ACSF (in B) or in Mg2+-free ACSF to activate NMDARs (in C) or in Mg2+-free ACSF in the presence of a combination of glycine, bicuculline and strychnine (in D), 20 min later, cells were harvested and processed for western blot analyses for P-pS6. Actin was used as loading control. Representative blots from 3–7 independent experiments per conditions are shown. Showing increased levels of P-pS6 (+ 111 ± 18% for high K+, n = 7; + 95 ± 13% for cLTP, n = 7). The effect on P-pS6 was suppressed by AP5 indicating that it was dependent on NMDARs and by rapamycin indicating increased activity of mTORC1. E) Incubation with BDNF (50 ng/mL) for 45 min in ACSF also increased the level of P-pS6 to a lower extent (+ 69 ± 20%, n = 4). F) Increased phosphorylation of Thr389-p70S6K in neurons stimulated in high-K+ saline as in (B). Shown is one blot out of two independent experiments. G) Inhibiting TrkB receptors with ANA12 reduced pS6 phosphorylation in neurons stimulated with high K+-ACSF as in (B). Shown is a representative western blot out of 3 independent experiments. H) Addition of either a cocktail of 20 amino acids (AA) or of only Leu, Gln and Arg for 15 min is also able to increase P-pS6 in neurons, as measured 15 min later by western blot (a representative western blot out of 2 independent experiments). I) DIV20 hippocampal neurons were treated for 10 min in ACSF containing TTX and AP5, with forskolin and rolipram or with dopamine with or without rapamycin (added 2 h before stimulation) and processed later for immunofluorescence to assess P-pS6 levels. Treatments aimed [file 13041_2021_820_MOESM1_ESM.pdf]

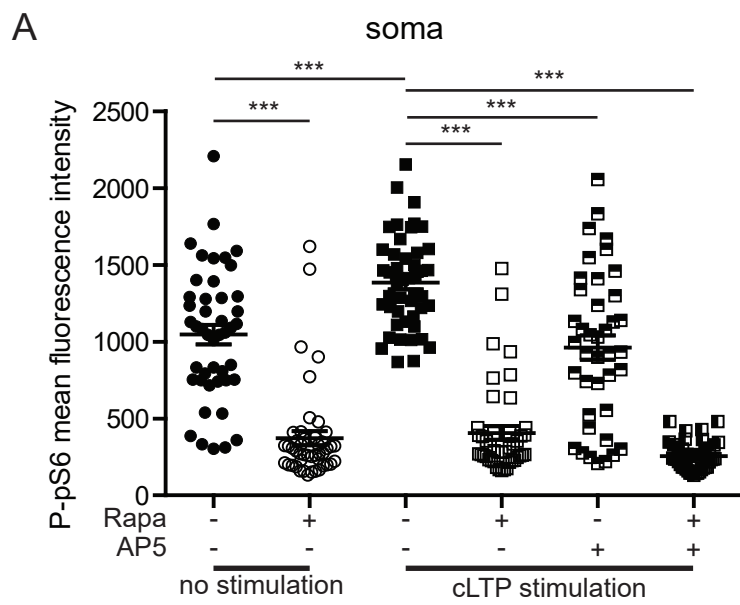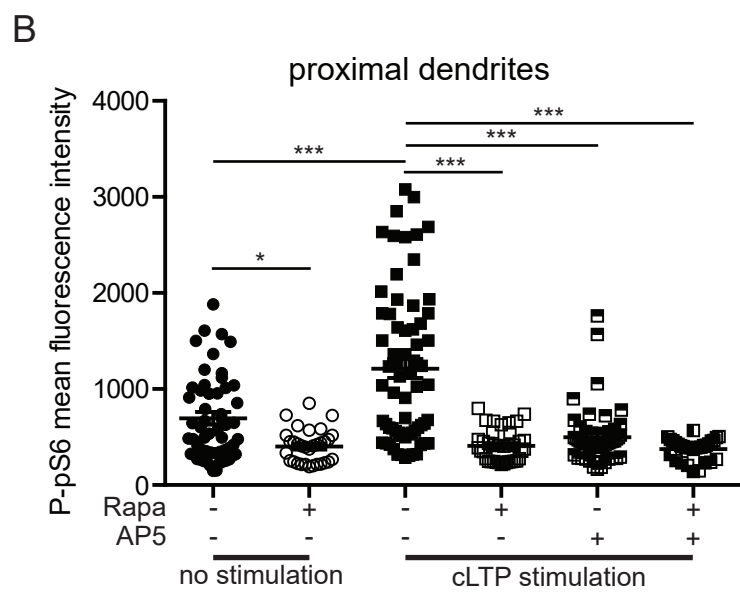

Supplement: Supplementary file 2 — Additional file 2: Figure S2. Increased pS6 phosphorylation 90 min after NMDAR stimulation in soma and proximal dendrites. Neurons were treated in cLTP medium as in Fig. 1B with or without AP5 or rapamycin as indicated, then incubated again in conditioned medium (CM) for 80 min before P-pS6 and MAP2 immunofluorescence. Note that over such extended-duration treatments, ACSF was replaced by CM to preserve neuron health. Individual neurons were imaged and P-pS6 quantified in soma or proximal dendrites (mean length 75 µM) for 40–60 neurons from 3 experiments. [file 13041_2021_820_MOESM2_ESM.pdf]

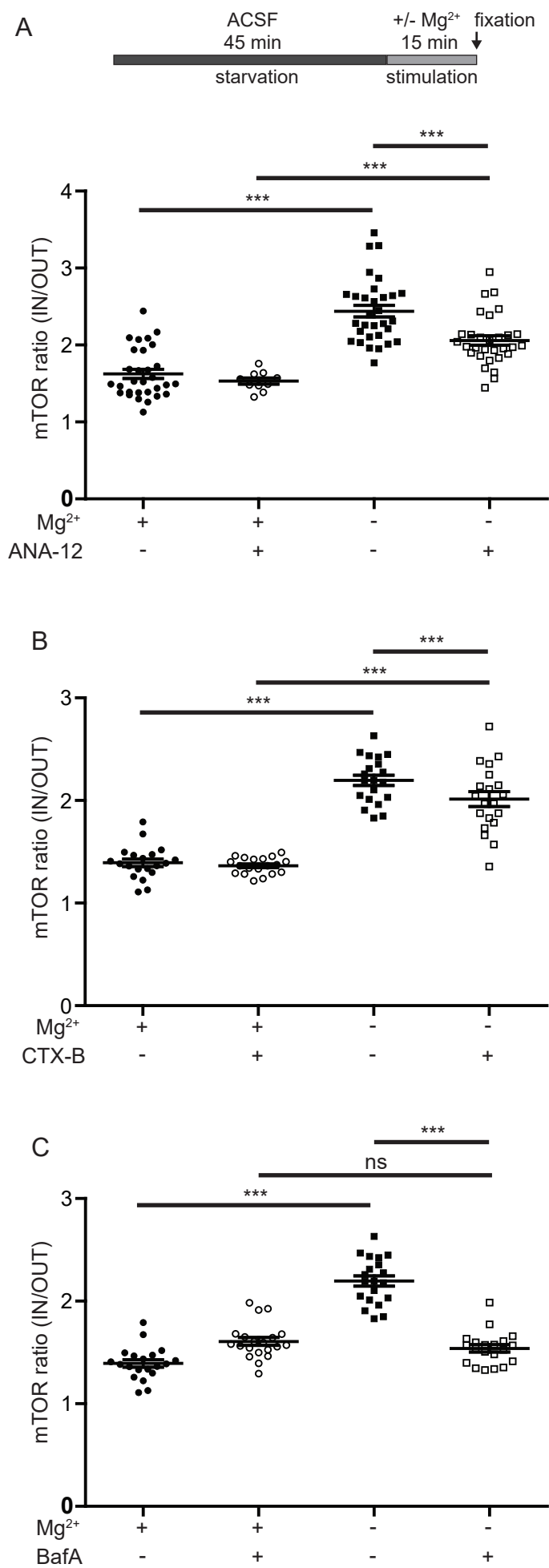

Additional figure 3

Supplement: Supplementary file 3 — Additional file 3: Figure S3. TrkB inhibitors ANA12 or cyclotraxin-B reduced the recruitment of mTOR to LEs (IN/OUT ratio, see Fig. 3) induced by NMDAR- activation even in starvation conditions. Neurons were treated as in Fig. 3D in ACSF plus Mg2+ or in Mg2+-free ACSF, as indicated A) Effect of ANA12 (25 µM) on mTOR IN/OUT ratio from 3 independent experiments. B) Effect of Cyclotraxin-B (CtxB) on mTOR IN/OUT ratio from 2 independent experiments. In stimulated neurons treated with the TrkB inhibitors, the IN/OUT mTOR ratio stays significantly higher than the one observed in non-stimulated neurons. C) Bafilomycin-A (BafA 200 nM) prevented the translocation of mTOR to LEs triggered by Mg2+ removal as in Fig. 3D (N = 20 images per condition from 2 independent experiments). [file 13041_2021_820_MOESM3_ESM.pdf]

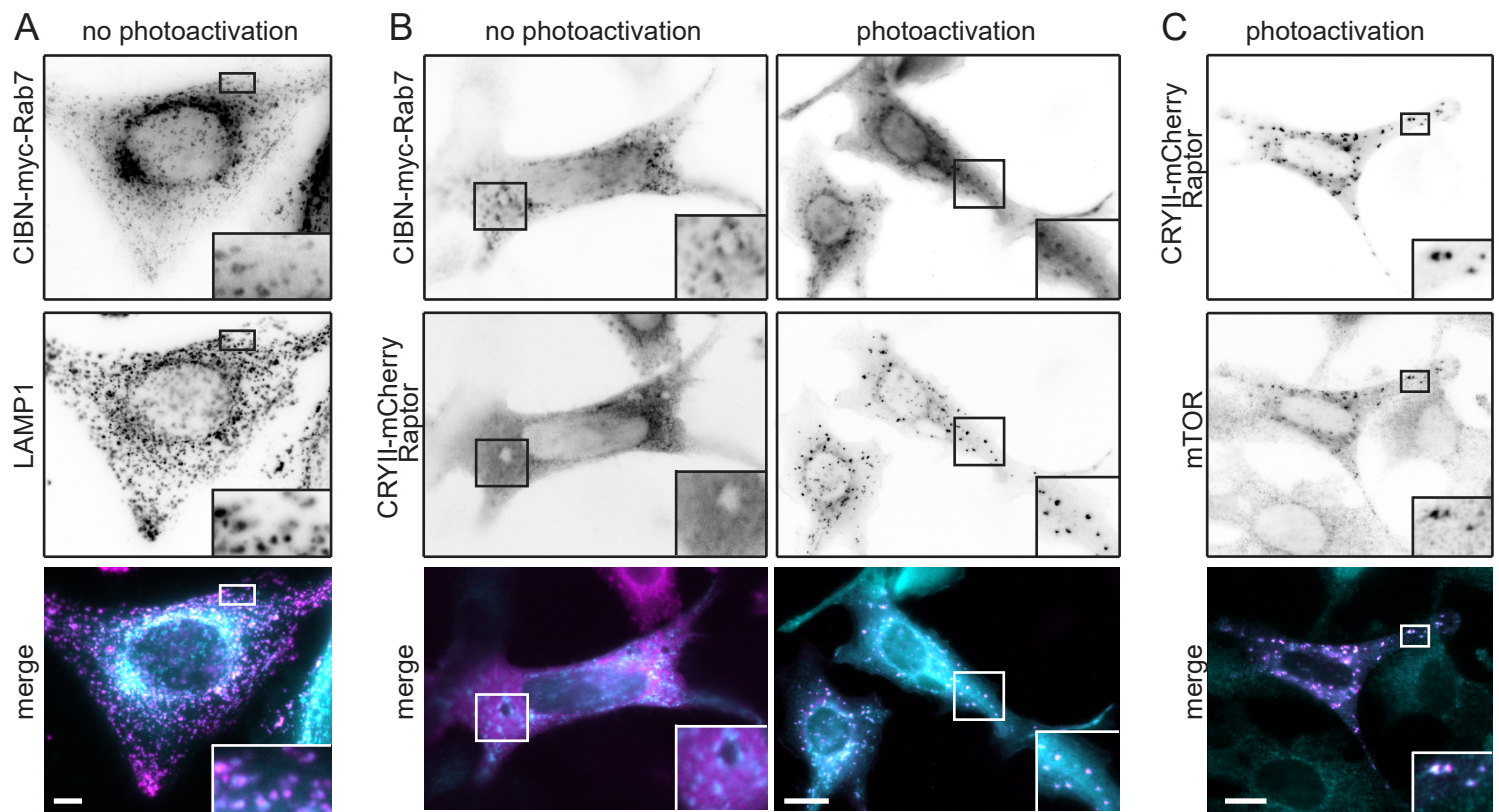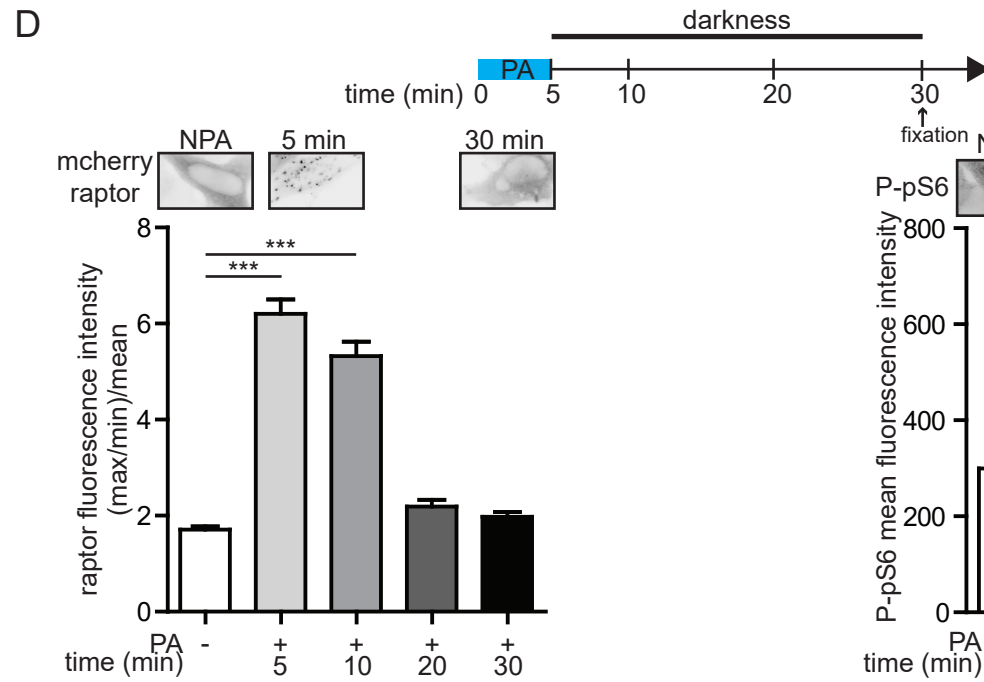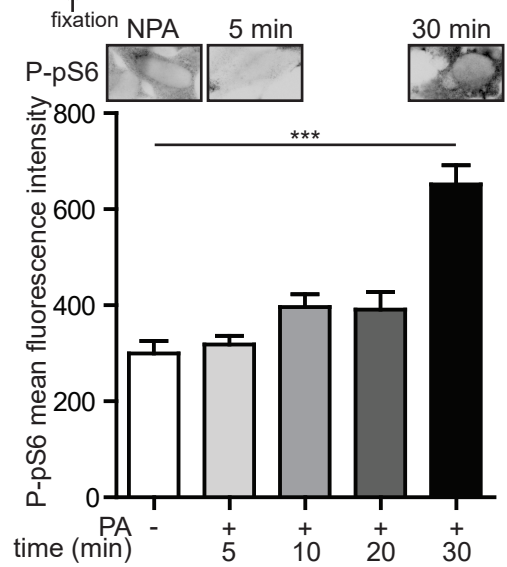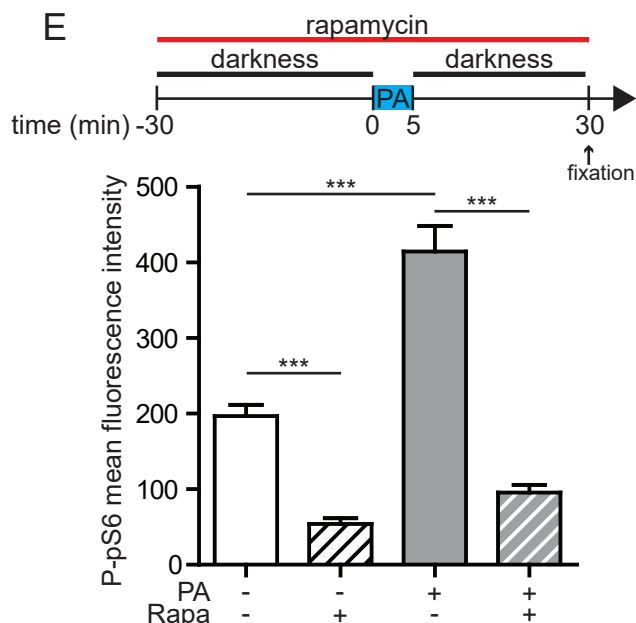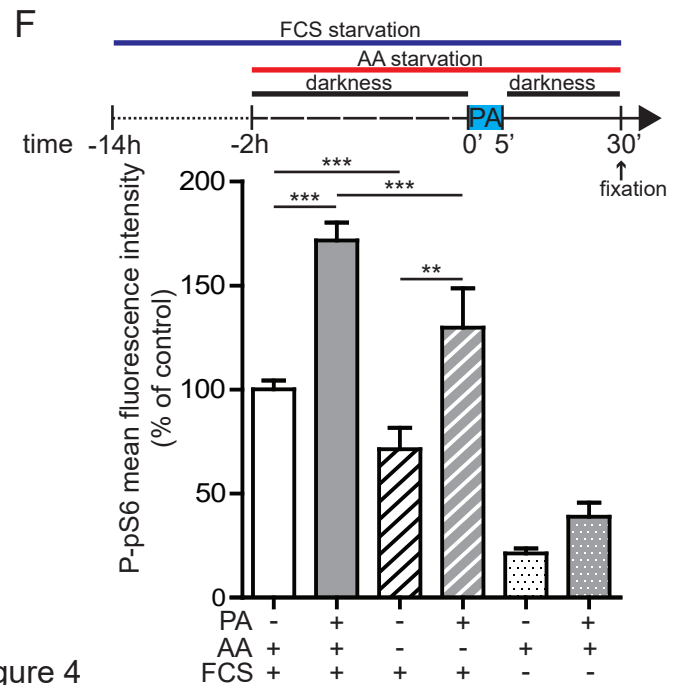

Additional figure 4

Supplement: Supplementary file 4 — Additional file 4: Figure S4. Set up of optogenetic activation of mTORC1 in HeLa cells. A) CIBN-Rab7 was correctly targeted to LEs in HeLa cells transfected with CIBN-myc-Rab7 and immunolabeled for LAMP1 and myc tag. B) Photoactivation was able to target CRY2-mCherry-Raptor to LEs. HeLa cells were transfected with CIBN-myc-Rab7 and CRYII-mCherry-Raptor; 24 h later cells were either kept in the dark (left panel) or photoactivated for 5 min (30 × 100 ms-pulses of blue light given at 0.1 Hz at 2.42 mW/cm2) (right panel) before being processed for immunofluorescence. C) Photoactivation (as in B) was able to target mTOR with CRY2-mCherry-Raptor to LEs. D) Left, clustering of CRY2-mCherry-Raptor on LEs induced by photoactivation reverses within a few min. Shown is an index of raptor clustering (intensity (max/min)/mean) per pixel obtained on raptor surface area). Right, the same treatment induces effective pS6 phosphorylation only 30 min after it was given. Experiments were performed as in (B) with immunofluorescence done at different times after illumination (PA) as indicated. Cells were incubated in culture medium after illumination (N = 90 images per condition from three independent experiments). E) Rapamycin blocks photoactivation-induced P-pS6 increase (N = 90 images per condition from three independent experiments). F) The effect of photoactivation-induced mTOR recruitment to LEs on mTOR activity depends on FCS but only partially relies on amino acids. Cells were fed ( +) or starved (-) with serum (FCS) or amino acids (AA) for the time indicated in the diagram. In AA-starved cells, photoactivation induces an increase in P-pS6, although significantly reduced in comparison to cells fed with both AA and FCS. FCS starvation suppresses mTORC1 activity even after photoactivation (N = 60 images per condition from two independent experiments). [file 13041_2021_820_MOESM4_ESM.pdf]
